# Adaptive optimization of the OXPHOS assembly line partially compensates lrpprc-dependent mitochondrial translation defects in mice

**DOI:** 10.1038/s42003-021-02492-5

**Published:** 2021-08-19

**Authors:** Alexanne Cuillerier, Matthieu Ruiz, Caroline Daneault, Anik Forest, Jenna Rossi, Goutham Vasam, George Cairns, Virgilio Cadete, The LSFC Consortium, Azadeh Aliskashani, Bruce G. Allen, Chantale Aubut, Chantal Bemeur, Claudine Beauchamp, Yan Burelle, Guy Charron, Lise Coderre, Christine Des Rosiers, Sonia Deschênes, François Labarthe, Jeannine Landry, Catherine Laprise, Geneviève Lavallée, Pierre Lavoie, Bruno Maranda, Charles Morin, Yvette Mukaneza, Tamiko Nishimura, John D. Rioux, Marie-Ève Rivard, Florin Sasarman, Eric A. Shoubridge, Jessica Tardif, Julie Thompson Legault, Nancy Tremblay, Vanessa Tremblay-Vaillancourt, Luc Vachon, Josée Villeneuve, Christine Des Rosiers, Yan Burelle

**Affiliations:** 1 Department of Cellular and Molecular Medicine, Faculty of Medicine, University of Ottawa, Ottawa, ON Canada; 2 Research Center, Montréal Heart Institute, Montréal, Québec Canada; 3 Department of Nutrition, Université de Montréal, Montréal, Québec Canada; 4 Interdisciplinary School of Health Sciences, Faculty of Health Sciences, University of Ottawa, Ottawa, ON Canada; 5 Sinclair Centre for Regenerative Medicine, Ottawa Hospital Research Institute, Ottawa, ON Canada; 6 Centre de santé et de services sociaux de Chicoutimi, Chicoutimi, Québec Canada; 7 Laboratoire Nutrition, Croissance et Cancer, Faculté de Médecine, Université de Tours, Tours, France; 8 Département des sciences fondamentales, Université du Québec à Chicoutimi, Chicoutimi, Québec Canada; 9 Association de l’acidose lactique du Saguenay-Lac-Saint-Jean, Québec, Canada; 10 Centre hospitalier universitaire de Sherbrooke, Sherbrooke, Québec, Canada; 11 Department of Human Genetics and Montreal Neurological Institute, McGill University, Montréal, Québec Canada

**Keywords:** Animal disease models, Biochemistry, Experimental models of disease

## Abstract

Mouse models of genetic mitochondrial disorders are generally used to understand specific molecular defects and their biochemical consequences, but rarely to map compensatory changes allowing survival. Here we took advantage of the extraordinary mitochondrial resilience of hepatic *Lrpprc* knockout mice to explore this question using native proteomics profiling and lipidomics. In these mice, low levels of the mtRNA binding protein LRPPRC induce a global mitochondrial translation defect and a severe reduction (>80%) in the assembly and activity of the electron transport chain (ETC) complex IV (CIV). Yet, animals show no signs of overt liver failure and capacity of the ETC is preserved. Beyond stimulation of mitochondrial biogenesis, results show that the abundance of mitoribosomes per unit of mitochondria is increased and proteostatic mechanisms are induced in presence of low LRPPRC levels to preserve a balance in the availability of mitochondrial- *vs* nuclear-encoded ETC subunits. At the level of individual organelles, a stabilization of residual CIV in supercomplexes (SCs) is observed, pointing to a role of these supramolecular arrangements in preserving ETC function. While the SC assembly factor COX7A2L could not contribute to the stabilization of CIV, important changes in membrane glycerophospholipid (GPL), most notably an increase in SC-stabilizing cardiolipins species (CLs), were observed along with an increased abundance of other supramolecular assemblies known to be stabilized by, and/or participate in CL metabolism. Together these data reveal a complex in vivo network of molecular adjustments involved in preserving mitochondrial integrity in energy consuming organs facing OXPHOS defects, which could be therapeutically exploited.

## Abstract (web summary)

Cuillerier et al. investigate compensatory mechanisms underlying survival of mice with a liver-specific knockout of the mitochondrial mRNA-binding protein Lrpprc. They propose various mechanisms operating along the OXPHOS assembly line, including mitochondrial biogenesis, mitochondrial ribosome upregulation and preferential supercomplex assembly, that could compensate lack of LRPPRC and allow survival of these mice.

## Introduction

Mitochondria are dynamic double-membrane entities playing a central role in cellular homeostasis. In addition to their major contribution to cellular energy production through oxidative phosphorylation (OXPHOS) these organelles are functionally coupled to peroxisomes and the endoplasmic reticulum and influence several facets of cellular biology such as lipid exchange, membrane dynamics, and signaling^1^. Because of their bacterial origin, each organelle is endowed with multiple copies of circular DNA (mtDNA) comprising a total of 37 genes encoding for 13 core components of the OXPHOS machinery, and all RNA components required for their translation. Over the course of evolution, the remaining genes essential for the synthesis of the 1158 mitochondrial proteins have however been transferred to the nucleus. As a result, the biogenesis of these organelles has become one of the most complex biological processes requiring fine intergenomic coordination, the import and assembly of protein subunits synthetized in distinct cellular compartments, and the incorporation of assembled multi-protein complexes into specialized lipid bilayers.

Not surprisingly, mutations in mitochondrial genes encoded either in the mtDNA or nucleus underlie a broad spectrum of inherited disorders^2–4^. A large proportion of these mutations affect structural subunits of complex I, III, IV, and V of the OXPHOS machinery, or factors involved in their translation or assembly. One such factor is Leucine-Rich PentatricoPeptide Repeat Containing protein (LRPPRC; OMIM*607544), a protein predominantly localized to the mitochondria involved in the stabilization and translation of mtDNA-endoded mRNAs. Mutations in the *Lrpprc* gene have been identified as the root cause of a distinct monogenic form of Leigh Syndrome in the French-Canadian population of the northeastern region of Quebec (Leigh Syndrome French Canadian variant; LSFC; OMIM#220111)^5,6^, and recently in unrelated families in Europe and China^7–9^. Mutations in *Lrpprc* result in a severe loss of the protein in tissues, and consequently a drastic reduction in the steady-state levels of most mitochondrial mRNAs, resulting in a pronounced complex IV (CIV) deficiency in the liver and brain (20% of normal activity), a moderate CIV deficiency in fibroblasts and heart (50% of normal activity) and a combined CIV and complex I (CI) defect in the skeletal muscle (40% of normal activity)^6,10,11^. LSFC patients also present high levels of circulating long-chain acyl-carnitines, which are proxies of mitochondrial fatty acid oxidation abnormalities, as well as a lipidomics signature of perturbations in peroxisomal lipid metabolism characterized by lower levels of circulating ether lipids, which are precursors of plasmalogens, as well as conjugated bile acids^12,13^.

Several features of the LSFC phenotype are also observed in mice harboring a liver-specific knockout of LRPPRC (*H-Lrpprc*^*−/−*^), which includes a growth delay, a depletion of most mtDNA encoded transcripts, a severe disruption of CIV assembly and activity, and an impairment of mitochondrial long-chain fatty acid oxidation and peroxisomal function^13,14^. However, mice behave normally and present striking preservation of respiratory chain capacity^14^. Moreover, their ability to tolerate fasting through the energetically costly process of gluconeogenesis is preserved, and they show no evidence of overt liver failure^14^, suggesting efficient adaptive changes are present to compensate for the translation defect resulting from LRPPRC deficiency. Better knowledge of those endogenous protective mechanisms has the potential to inform novel and unsuspected opportunities for therapeutic interventions, which are currently lacking for these patients.

In this study, native proteomics profiling and lipidomics in mitochondrial membranes were therefore used in our well-characterized mouse model of hepatic LRPPRC deficiency to explore and integrate various aspects that are critical for the maintenance of mitochondrial bioenergetics integrity. Our results point to several adaptive countermeasures which collectively allow for relative preservation of mitochondrial and liver function despite the severity of the LRPPRC-dependent translation defect.

## Results

### Hepatic Lrpprc deficiency triggers compensatory mitochondrial biogenesis and increased abundance of mitoribosome complexes

**Fig. 1:**
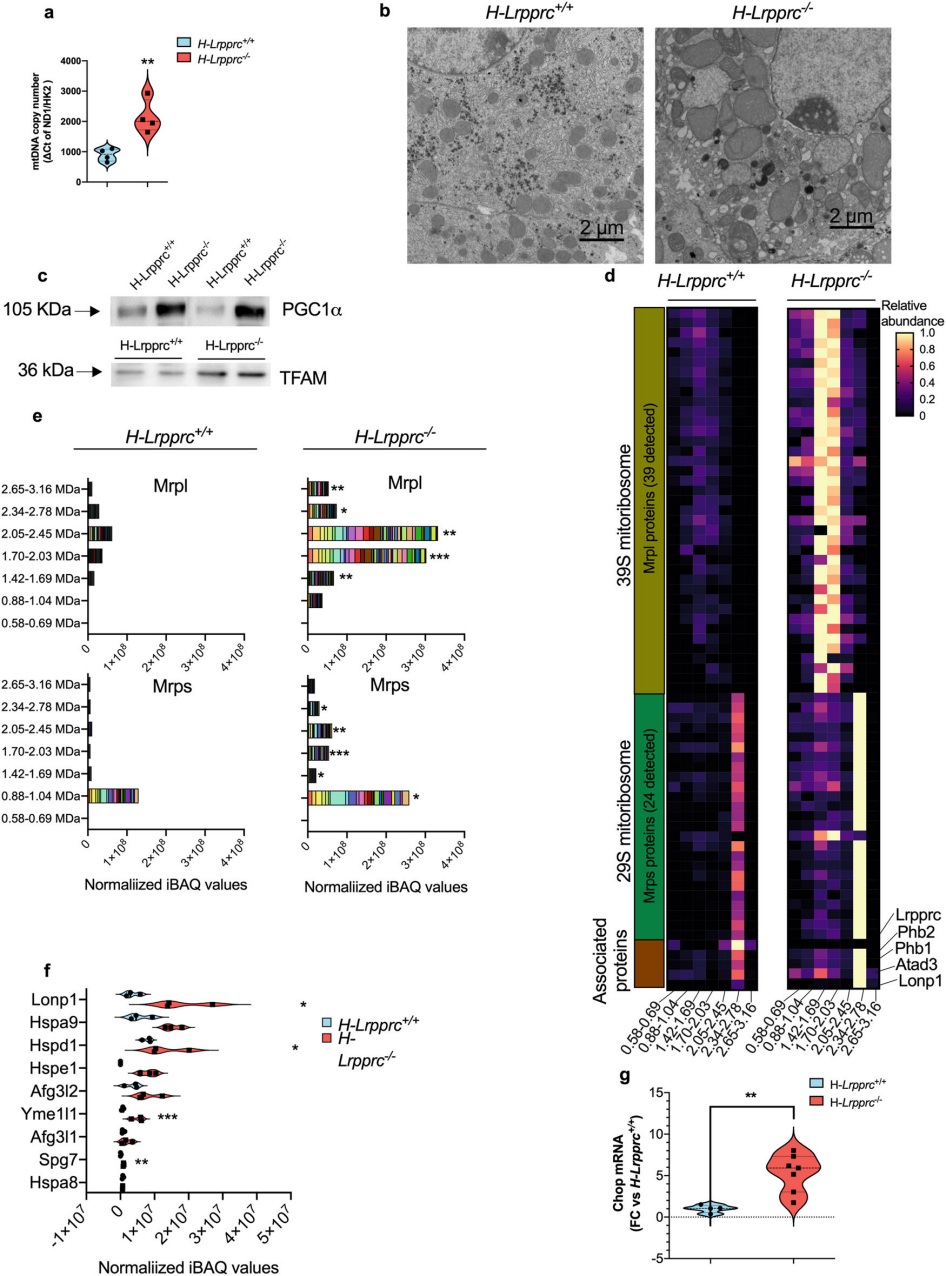
Impact of LRPPRC deficiency on mitochondrial biogenesis, mitochondrial ribosome content, and proteostatic systems. **a** mitochondrial DNA copy number measured in whole liver homogenates from of *H-Lrpprc*^*+/+*^ and *H-Lrpprc*^*−/−*^ mice at 5 weeks of age. Values represent means ± sem of the ND1/HK2 ΔCt ratio (*n* = 4). Student *T*-test was used to assess significance (**P < 0.01). **b** Transmission Electron Micrographs of *H-Lrpprc*^*+/+*^ and *H-Lrpprc*^*−/−*^ livers at 5 weeks of age (magnification 1900x). **c** PGC1α and TFAM protein expression in whole liver homogenates from *H-Lrpprc*^*+/+*^ and *H-Lrpprc*^*−/−*^ mice. **d**, **e** Proteomic profiling of Large 39 S and Small 29 S mitochondrial ribosomal proteins (MRPL and MRPS proteins) in digitonin-solubilized mitochondria from *H-Lrpprc*^*+/+*^ and *H-Lrpprc*^*−/−*^ mice following separation by hybrid CN/BN-PAGE. The heatmap (**d**) represents the average abundance (*n* = 3 per group) of individual ribosomal protein in each of the gel bands analyzed relative to the maximal abundance observed for this protein in all samples analyzed. Each lane represents an individual subunit, and the range of molecular weight covered by each band is indicated at the bottom. To represent the abundance of mitoribosomes, iBAQ intensities for all of the large and small ribosomal subunits detected in each band were summed for each experimental replicate and the average values obtained for each genotype is presented in stacked histograms (**e**). **f** Total abundance of selected mitochondrial proteases in digitonin-solubilized mitochondria from *H-Lrpprc*^*+/+*^ and *H-Lrpprc*^*−/−*^ mice. For a given protein, total abundance in a sample was calculated by summing the normalized iBAQ intensities observed in each band analyzed. Values presented represent the mean ± sem for *n* = 3 in each group. Validation immunoblots for selected proteases are shown in Fig. S1. One sample *T* test corrected for multiple comparisons were performed to assess significance (***Q < 0.01, **Q < 0.01, * Q < 0.05). **g** transcript level of *Chop* normalized to *Tbp* as a housekeeping gene in whole liver homogenates from H-*Lrpprc*^+/+^ (*n* = 4) and H-*Lrpprc*^*−/−*^ (*n* = 7).

Activation of mitochondrial biogenesis to compensate specific biochemical defects has been reported previously in patients with mitochondrial disorders caused by mtDNA mutations, particularly when the heteroplasmy threshold reaches 60–90%^15,16^. To begin our investigation, we therefore examined whether this common adaptive mechanism was present in livers from *H-Lrpprc*^*−/−*^ mice. As shown in Fig. 1a, copy number was more than doubled compared to WT controls, and mitochondria, although presenting clear ultrastructural abnormalities, were visibly larger and more numerous when visualized by electron microscopy (Fig. 1b). Furthermore, protein expression levels for PGC1α, and the transcription factor TFAM were also upregulated, consistent with an increased drive for mitochondrial biogenesis in presence of low levels of LRPPRC (Fig. 1c).

In mitochondria, the main role of LRPPRC is to act as a global RNA chaperone that binds and stabilizes mt-DNA-encoded RNA structures to promote their translation^10,17^. Therefore, we examined whether quantitative adjustments in the translational machinery were also present to compensate for the reduced levels of mitochondrial transcripts observed in absence of LRPPRC. To this end, proteomics profiling was performed on digitonin-extracted mitochondrial samples submitted to hybrid CN/BN-PAGE to resolve and quantify the abundance of mitochondrial ribosome complexes. A total of 24 Small and 39 Large Ribosomal Proteins (MRPS and MRPL) were consistently detected in all samples. In both genotypes, peak abundances for MRPS and MRPL proteins were observed in the 0.88–1.04, and 2.05–2.45 MDa regions respectively, which correspond to the migration pattern of the 28 and 39 S mitoribosome complexes^18^ (Fig. 1d, e). The global abundance of MRPS and MRPL proteins in both of these complexes was increased several folds in LRPPRC deficient mitochondria. Because equal amounts of mitochondrial protein extracts were loaded in the gels, these results suggest that the number of mitoribosomes per mitochondrial unit was substantially increased (Fig. 1d, e). Of note, LRPPRC was only detected in WT mitochondria and was mostly associated with the 28 S mitoribosome complex together with prohibitins (PHB1, PHB2), and ATAD3, which are known mitoribosome-associated proteins (Fig. 1d). Altogether, these results indicate that in addition to a greater cellular mitochondrial content, the density of ribosomes in each mitochondria is enhanced in *H-Lrpprc*^*−/−*^ mice in order to compensate for the translation deficit.

Proteomic profiling also revealed that several proteins involved in the Mitochondrial Unfolded Protein Response (mtUPR) were increased in response to the LRPPRC deficit, including mtHSP70/GRP75 (Hspa9), HSP60 (Hspa1), HSP10 (Hspe1), LONP1, AFG3L2, SPG7, and YME1L1 (Fig. 1f). Several of these proteins were also found to be upregulated in confirmatory immunoblot experiments (Fig. S1). Furthermore, transcript levels for the transcription factor CHOP, which mediates the mtUPR and more broadly the Integrated Stress Response^19^, was increased 5-fold in *H-Lrpprc*^*−/−*^ livers (Fig. 1g), suggesting rewiring of cellular metabolism and maintenance of mito-nuclear balance likely involves these survival pathways.

### Hepatic Lrpprc deficiency triggers a remodeling of respiratory chain supercomplexes and stabilization of residual CIV into respirasomes

Studies on isolated mitochondria from *H-Lrpprc*^*−/−*^ mice showed that maximal uncoupled flux through the electron transport chain (ETC) is completely preserved despite a drastic reduction (80–85%) in the amount of assembled CIV and in enzyme activity measured in solubilized extracts^14^. Even more striking is the fact that respiration in presence of the CIV substrate TMPD/ascorbate, which reflects CIV activity in its native membrane environment, is only reduced by 20–30%, as opposed to 80–85% following detergent extraction (^14^ and Fig. S2). These results strongly suggest that beyond the above-mentioned increase in mitochondrial biogenesis and mitoribosome density, additional mechanisms are in place to preserve residual CIV activity and maintain ETC function in presence of low LRPPRC levels.

**Fig. 2:**
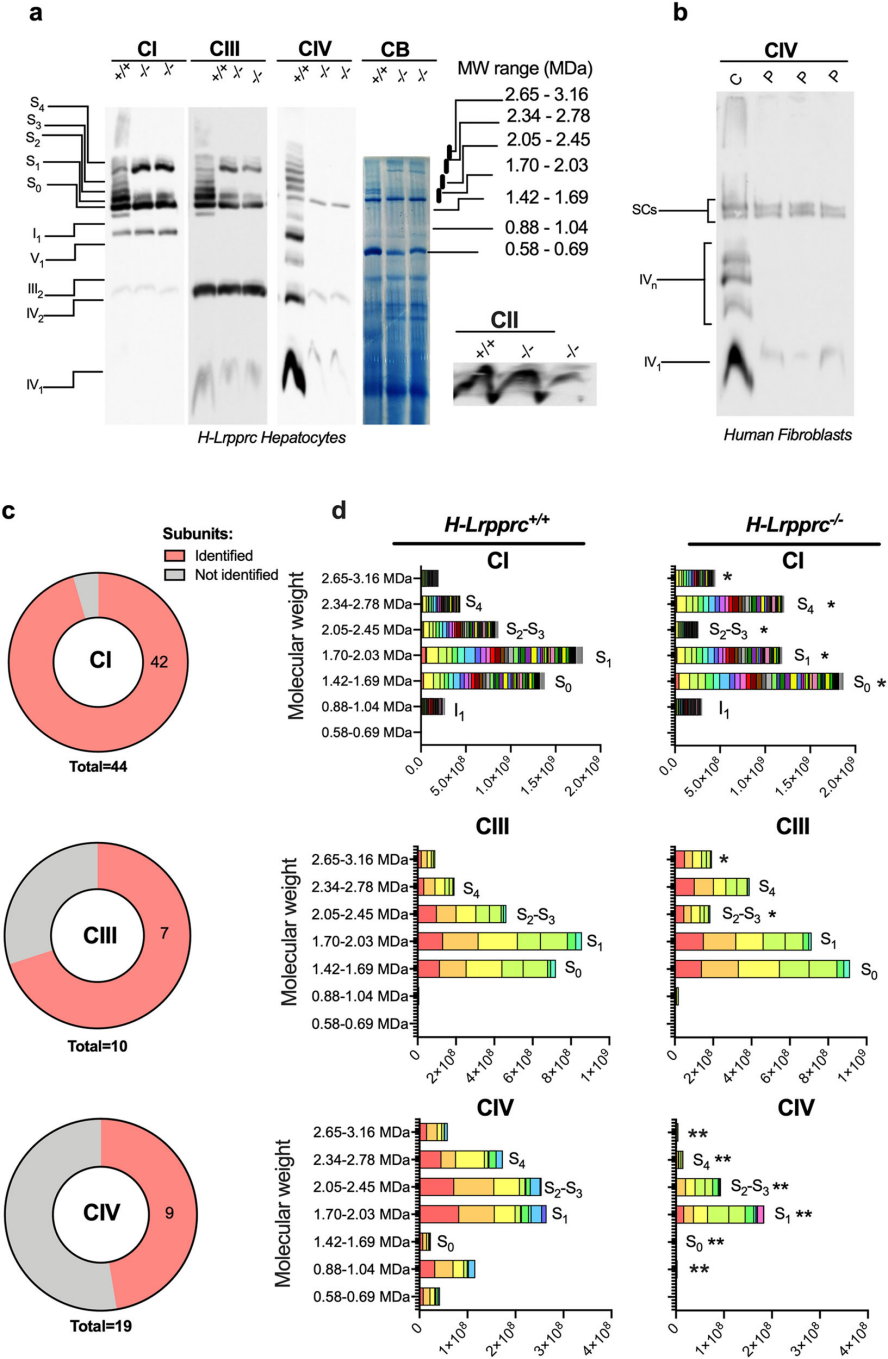
Impact of LRPPRC deficiency on respiratory chain supercomplexes. **a** Representative migration pattern of respiratory chain supercomplexes in digitonin-solubilized mitochondrial extracts from *H-Lrpprc*^*+/+*^ and *H-Lrpprc*^*−/−*^ mice resolved by hybrid CN/BN-PAGE. Replicates of the same 3 samples (1 *H-Lrpprc*^*+/+*^ and 2 different *H-Lrpprc*^*−/−*^) were loaded in multiple wells and migrated together. Following electrotransfer, replicate lanes were cut and probed with specific antibodies for CI, CII, CIII, and CIV. OXPHOS complexes and supramolecular assemblies are identified using the standard nomenclature, with numbers in indices indicating the molecular stoichiometry of each OXPHOS complex. Molecular weight ranges indicated on the right correspond to the size of the bands that were excised from a gel ran in parallel for proteomics profiling. Molecular weight calibration was performed using the migration pattern of CI, CII, CIV, and CV as assessed by in-gel activity assays. **b** Representative migration pattern of CIV-containing supercomplexes in fibroblast mitochondria from controls and LSFC patients revealed by CIV immunostaining. Electrophoresis conditions were similar as in panel (**a**). **c** Quantitative analysis of the proportion of CIV present in SCs relative to monomeric CIV in liver mitochondria and patient fibroblasts (*n* = 3 per group). **d** Proteomics analysis of respiratory chain supercomplexes. For each complex, a pie chart illustrates the number of subunits reliably identified in the gel bands and the stacked histogram illustrates the mean (*n* = 3 per group) abundance of complex subunits present in each band analyzed (see Fig. 1f legend for details).

We therefore tested whether the preservation of residual CIV was linked to remodeling of the ETC supercomplexes (SCs). Digitonin-solubilized mitochondria were subjected to hybrid CN/BN-PAGE, and the various supramolecular arrangements of ETC complexes were examined by immunoblot (Fig. 2a), in-gel activity (Fig. S3), and proteomics profiling (Fig. 2d). As expected, the total amount of assembled monomeric CIV was considerably reduced in LRPPRC deficient mitochondria compared to WT, with no apparent changes in the amount of CI monomers and CIII dimers (Fig. 2a). However, incorporation of CIV monomers into the S_1_ supercomplex composed of monomeric CI, dimeric CIII, and monomeric CIV (I_1_-III_2_-IV_1_, termed respirasome) was relatively preserved (Fig. 2a). As a result, the proportion of CIV incorporated in the S_1_ respirasome relative to its monomeric form was increased (Fig. 2c, top, and S3). Since only a few CIV monomers are available for assembly, higher molecular weight supercomplexes containing dimeric and multimetric CIV (e.g. S_2_-S_4_: I_1_-III_2_-IV_2_ and I_1_-III_2_-IV_n_) were nevertheless severely reduced in *H-Lrpprc*^*−/−*^ mitochondria (Fig. 2a), albeit still detectable by proteomic analysis of the bands (Fig. 2d), and in-gel activity measurement for a prolonged incubation period (Fig. S3). LRPPRC deficient mitochondria also presented a strong accumulation of CI and CIII in the region corresponding to the S_4_ supercomplex despite containing almost no CIV (Fig. 2a, d). This was accompanied by a reduction of CI and CIII in the region corresponding to S_2_ and S_3_ supercomplexes (Fig. 2a, d and S3) in *H-Lrpprc*^*−/−*^ mitochondria, indicating a migration shift of CI-CIII containing arrangements toward a higher apparent molecular weight. Furthermore, the S_0_ supercomplex composed of monomeric CI and dimeric CIII (I-III_2_) was slightly but significantly more abundant in *H-Lrpprc*^*−/−*^ mitochondria (Fig. 2d).

SCs were also extracted from LSFC patient fibroblasts and resolved under the same electrophoresis conditions. As shown in Fig. 2b, c (bottom), the abundance of CIV monomers was also severely reduced in LSFC patient mitochondria compared to control. However, the abundance of CIV present in S_1_-S_2_ SCs was relatively preserved, confirming the stabilization of residual CIV into supramolecular assemblies in these LSFC patient cells. Oligomeric forms of CIV found in control cells between the monomeric band and SCs assemblies, were however undetectable in patient samples. Stabilization of residual CIV in supercomplexes has also been reported in fibroblasts from two patients harboring isolated CIV deficiencies linked to mutation of distinct nuclear-encoded genes^20^, suggesting this may be a common response to CIV deficiency.

### Integration of residual CIV in respirasomes in LRPPRC deficient mitochondria is independent of COX7A2L

The molecular mechanisms regulating the formation of SCs are currently unclear and constitute a topic of debate^21^. Several proteins, including COX7A2L, RCF1/HIGD1A, and RCF2/HIGD2A, have been identified using knockdown approaches in yeast and mammalian cells, and are suggested to affect SCs. However, controversy exists regarding their necessity, and whether they act directly in the formation/stability of SCs, or indirectly by promoting the assembly of individual ETC complexes.

**Fig. 3:**
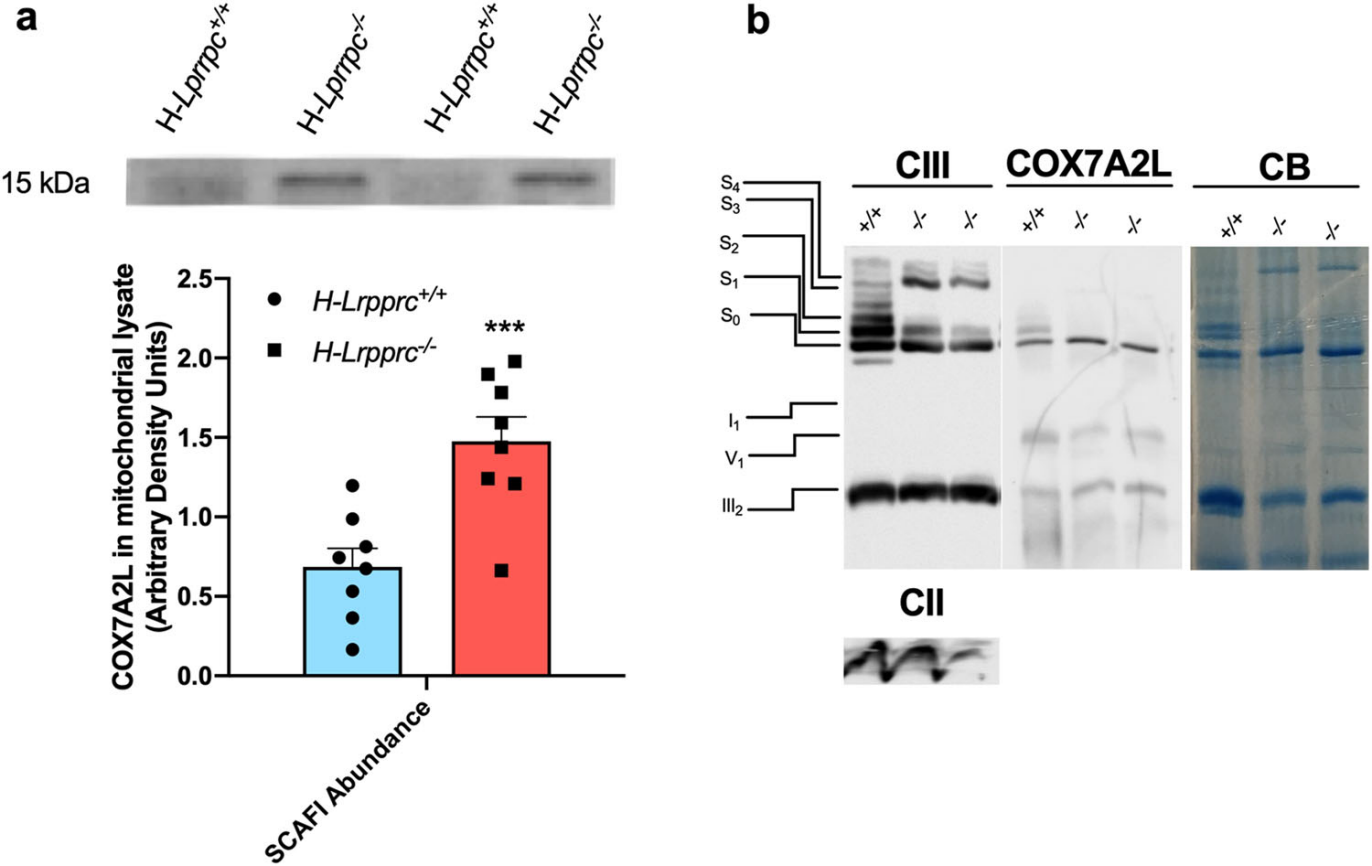
Impact of LRPPRC deficiency on supercomplexes assembly factor SCAF1. **a** Western blot for quantification of SCAF1 abundance in liver mitochondria lysate (*n* = 8 per group) with a representative image of the blot. (**p < 0.01, Unpaired *t*-test). **b** Representative migration pattern of CIV containing arrangements from digitonin solubilized extracts from *H-Lrpprc*^*+/+*^ and *H-Lrpprc*^*−/−*^ mice resolved by hybrid CN/BN-PAGE (*n* = 2 per group). After electrotransfer, one replicate was probed with antibody for CIII and CII, and the other replicate of the same gel with antibody for SCAF1 (COX7A2L).

As shown in Fig. 3a, *Lrpprc* deficiency caused a 2 fold increase in total COX7A2L protein abundance in whole mitochondrial lysates. When resolved under native conditions, COX7A2L was found mainly in CIII dimers and I_1_-III_2_ assemblies where its abundance was increased by ~1.5–2.0 fold in H-*Lrpprc*^*−/−*^ samples (Fig. 3b). However, COX7A2L was virtually absent from the respirasome and the larger molecular weight SCs (Fig. 3b), ruling out a potential contribution of COX7A2L to the stabilization of CIV into SCs observed in *Lrpprc*-deficient mitochondria. This observation is consistent with the fact that mice were on a C57BL/6 J background and thus expressed the short variant of COX7A2L which is able to interact with CIII assemblies, but lacks 2 critical residues found to mediate interactions with CIV^22–25^. Of note, HIGD1A and HIGD2A (the mammalian orthologs of yeast’s RCF1 and RCF2), which were recently suggested to promote SC assembly^26–28^, could not be detected by proteomics profiling of the various SC bands (Supplemental Data 1). Overall, our results suggested the possibility that factors other than assembly proteins could underlie the stabilization of CIV into SCs in *Lrpprc*-deficient mitochondria.

### Hepatic Lrpprc deficiency induces a remodeling of mitochondrial glycerophospholipids and increases multiple cardiolipin species

Beyond assembly factors, increasing evidence indicates that membrane glycerophospholipid (GPL) composition exerts a strong influence on the assembly/stability of multi-protein complexes^29,30^. Thus, comprehensive untargeted lipidomics profiling, as previously described^13,31^, was performed in the digitonin-extracts used for hybrid CN/BN-PAGE in order to characterize potential changes in phospholipids that could promote SC stability in LRPPRC deficient mitochondria. Prior to performing these experiments, we validated the digitonin concentration used had a negligible impact on the GPL profile in the protein extract in order to exclude any biases related to small variations in the digitonin concentration across samples (Fig. S4). No significant changes in the proportion of the GPL (phosphatidylethanolamine and lysophosphatidylethanolamine (PE; LPE) shown as examples) and CL detected was noted between samples extracted with a digitonin ratio of 2 or 4 g/g of proteins.

**Fig. 4:**
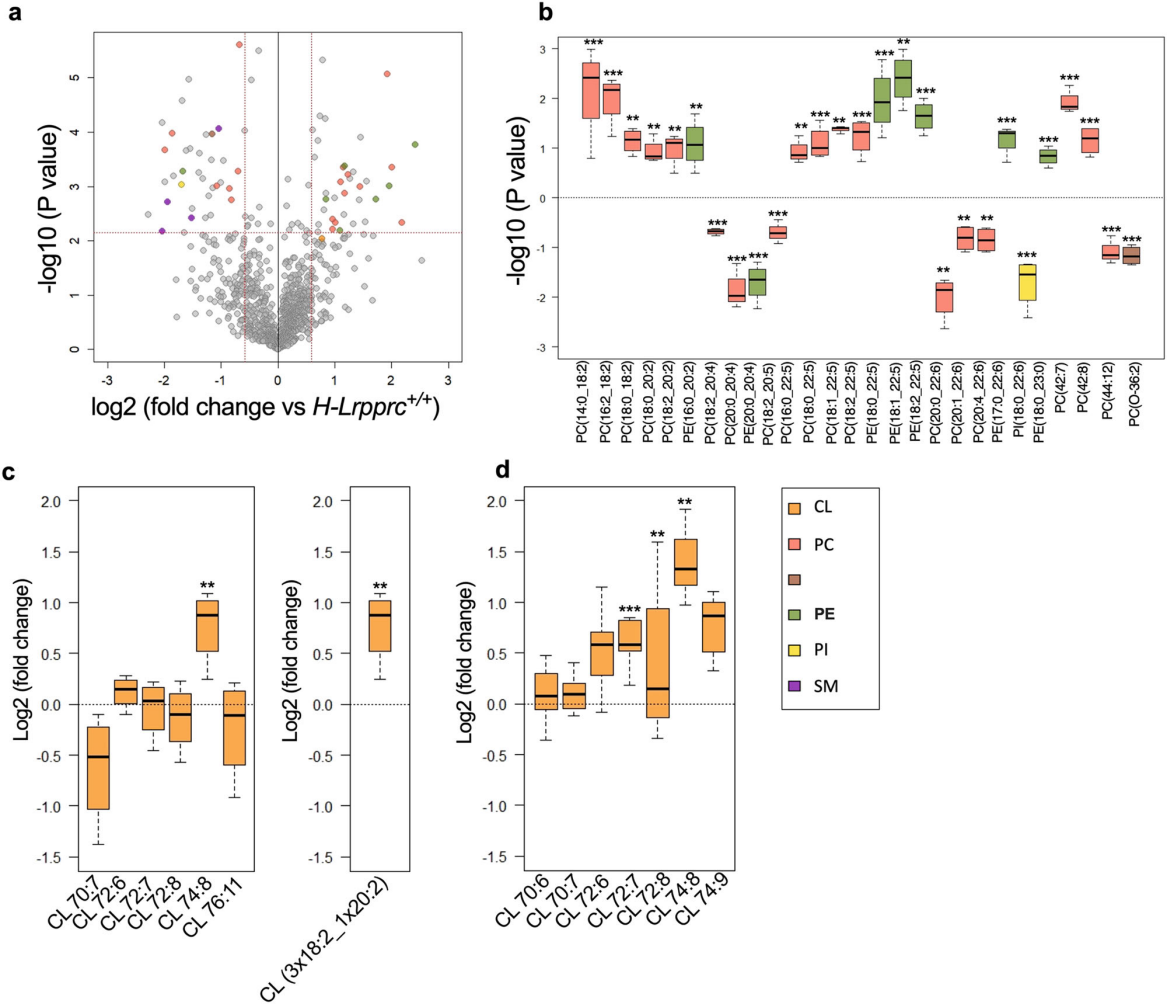
Impact of LRPPRC deficiency on mitochondrial membrane lipids. Comprehensive lipidomics of digitonin solubilized mitochondrial extracts (**a**, **b**) and targeted data mining of cardiolipin (CL) in digitonin-extracts (**c**) or isolated mitochondria (**d**). **a** Volcano plot from LC-QTOF-based untargeted lipidomics of digitonin solubilized mitochondria from H-*Lrpprc*^+/+^ and H-*Lrpprc*^*−/−*^ (*n* = 4 per group). The *x* axis represents fold changes (FCs) of MS signal intensity values for all these features in H-*Lrpprc*^*−/−*^ vs. H-*Lrpprc*^+/+^ (log2) and the y axis represents *p* values (−log10). A corrected *p* value (*Q*-value) threshold of 0.05 (corresponding to a *P*-value of 0.007) and a relative FC of 1.5 were used, leading to 69 significantly discriminant features among the 1176 MS features on which 31 were annotated. **b** Box plot of 27 selected lipids significantly discriminating H-*Lrpprc*^*−/−*^ from H-*Lrpprc*^+/+^, and annotated using MS/MS analysis. The midline represents the median fold change vs. *H-Lrpprc*^*+/+*^, the box represents the interquartile range (IQR) between the first and third quartile, and whiskers represent the lowest or highest values. **c**, **d** Box plot of CL species annotated by MS/MS analysis in the digitonin-extracts (**c**) and in isolated mitochondria (**d**). Differences are shown according to a *Q* value of 0.056 (corresponding to a *P*-value of 0.009); (***P < 0.01, **P < 0.01).

In the digitonin-extracts, a total of 1176 mass spectrometry (MS) signals or features were reliably detected using high-resolution Liquid Chromatography quadrupole time-of-flight (LC-QTOF). These MS signals include, among other lipids, phosphatidylcholines (PCs), and the non-bilayer forming GPL phosphatidylethanolamines (PEs) as well as cardiolipin (CLs), which together make up ~90% of total mitochondrial GPL^29^. Volcano plot representation of the data highlighted the most significant changes, using a *Q*-value of < 0.05 (corresponding to *P* < 0.007) and a relative fold change (FC) threshold of 1.5 and 0.75, in mitochondrial membrane lipids from H-*Lrpprc*^*−/−*^ compared to H-*Lrpprc*^+/+^ mice (Fig. 4a). MS/MS analysis of the most discriminant entities revealed changes in all the three major classes of mitochondrial GPL including PCs, PEs, and CLs (Fig. 4b). All of the differentially abundant PEs detected, except one (PE(20:0_20:4)), were increased in H-*Lrpprc*^*−/−*^ mitochondria. For PCs, the proportion of increased and decreased species in H-*Lrpprc*^*−/−*^ mice was more balanced; but interestingly those that were decreased were mostly enriched in DHA (22:6) in the sn-2 position (Fig. 4b). In addition to these changes, four sphingomyelins were also found to be significantly less abundant in LRPPRC deficient extracts (SM(d18:1/22:0): FC 0.26, *Q* = 0.0217; SM(d18:1/20:4): FC 0.35, *Q* = 0.0358; SM(d18:2/18:1): FC 0.48, *Q* = 0.0047; SM(d40:2): FC 0.24, *Q* = 0.049)(Fig. 4a).

CLs being the mitochondrial GPL for which a role in SC assembly, stability, and function has been established^32–35^, a targeted data mining approach was used to quantify all detectable CLs in the digitonin-extracts. Six species that collectively account for > 90% of the total CL pool in the liver^36^ were consistently identified in the digitonin-extracts (Fig. 4c). This included the symmetric CL species CL72:6, CL72:7, and CL72:8 which account for ~80% of the total CL pool^36^, as well as less common asymmetric species (CL 70:7, CL 74:8 and CL 76:11: ~10% of total CL pool^36^,) in which one C18:2 acyl chain is substituted by either hexadecadienoic acid (C16:2), eicosadienoic acid (C20:2) or docosapentaenoic acid (C22:5) (Fig. 4c). Of those, only CL 74:8 was significantly more abundant H-*Lrpprc*^*−/−*^ mitochondria (log2 FC: 0.9, *Q* 0.05) and MS/MS analysis confirmed the acyl chains composition (CL(18:2_18:2_18:2_20:2)). However, broader changes in the CL profile were observed in whole mitochondrial lysates, with four species being significantly more abundant in H-*Lrpprc*^*−/−*^ mitochondria (Log2 FC 0.6–1.2; *Q* < 0.05), namely CL 72:6, and CL 72:7 which account for ~30% of the total CL pool^36^, as well as CL 74:8 and CL 74:9 (Fig. 4d). Collectively, these results indicate that LRPPRC deficiency leads to significant remodeling of the GPL landscape in mitochondria, including a relative increase in PE species compared to PC and an increase in the abundance of multiple CLs species, which may promote stabilization of residual CIV into SCs.

### Hepatic Lrpprc-deficiency is associated with changes in protein complexes that are structurally and functionally linked to CL

**Fig. 5:**
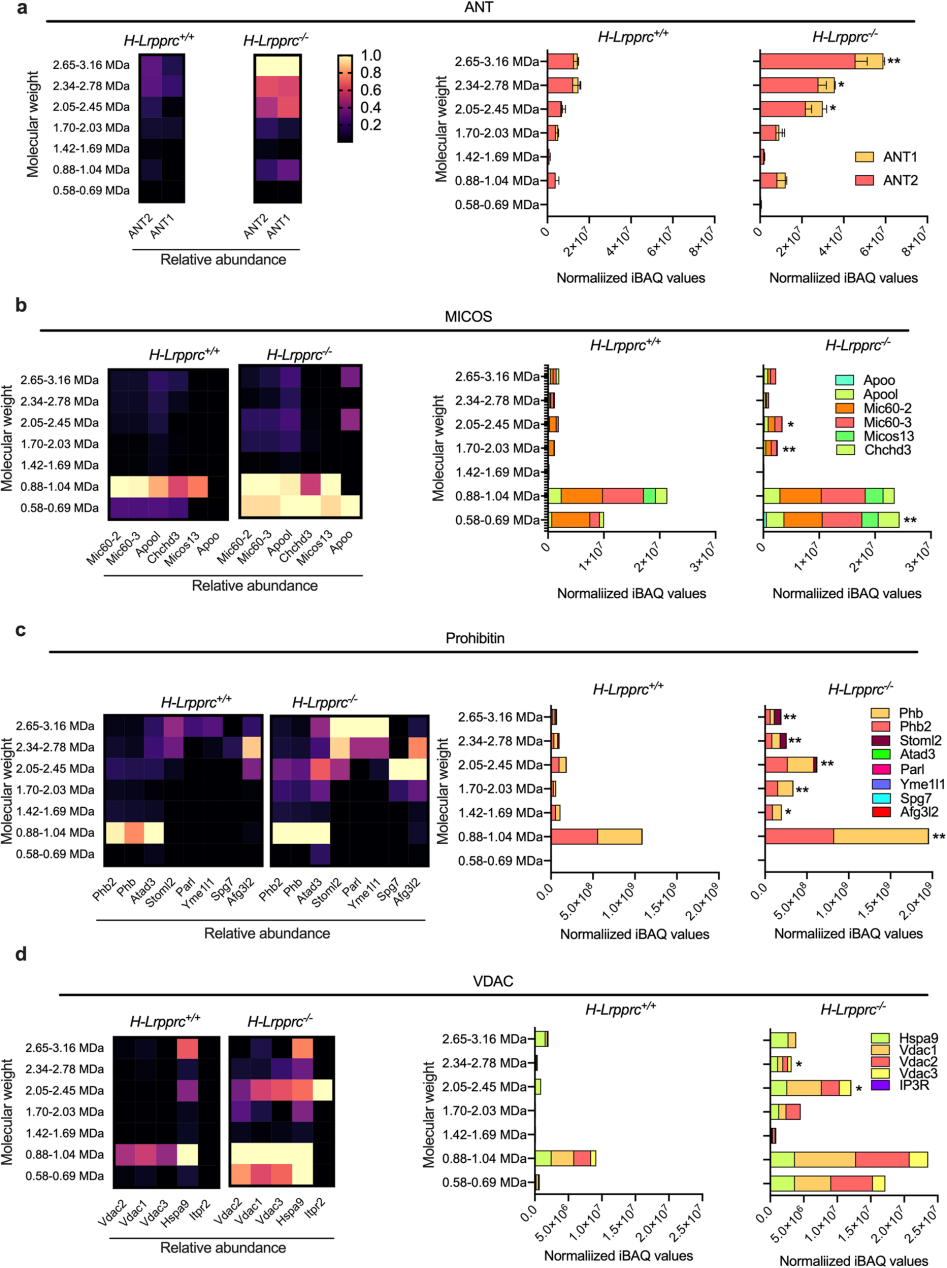
Impact of LRPPRC deficiency on protein complexes structurally and functionally linked to CL. Proteomic profiling of ANT (**a**), MICOS (**b**), Prohibitin (**c**), and VDAC (**d**) complexes in digitonin-solubilized mitochondria from *H-Lrpprc*^*+/+*^ and *H-Lrpprc*^*−/−*^ mice following separation by hybrid CN/BN-PAGE. The heatmap represents the average abundance (*n* = 3 per group) of individual protein within the complex in each of the gel bands analyzed relative to the maximal abundance observed for this protein in all samples analyzed. Each column represents an individual subunit, and the range of molecular weight covered by each band is indicated on the left side. To represent the abundance of ANT, MICOS, Prohibitin, and VDAC, iBAQ intensities for all of the listed proteins forming each complex detected in each band were summed for each experimental replicate and the average values obtained for each genotype is presented in stacked histograms. Values represent the mean ± sem for *n* = 3 in each group. One sample *T* tests corrected for multiple comparisons were performed to assess significance (***Q < 0.01, **Q < 0.01, *Q  < 0.05).

To further explore a potential role for increased CLs in promoting SC stability, native gel proteomics was used to probe selected supra-molecular complexes reported to be stabilized by CLs, or involved in the formation of CLs microdomains. To this end, we first determined whether the association of the ATP/ADP exchanger (ANT) with SCs was enhanced in *H-Lrpprc*^*−/−*^ mitochondria, since the association of a minor fraction of ANT to SCs was previously shown to be strictly dependent on CLs^37^. As shown in Fig. 5a, ANT1 and ANT2 were both detected in high molecular weight bands corresponding to SCs, and their abundance was 2–3 folds greater in *H-Lrpprc*^*−/−*^ mitochondria compared to WT, despite the fact that equal amounts of mitochondrial protein extracts were loaded in the gels. Similarly, CLs are involved in the oligomerization and stabilization of MICOS complexes in the inner mitochondrial membrane where they participate in the importation of precursors for CL synthesis and the formation of CL-rich microdomains^38,39^. As shown in Fig. 5b, MICOS subunits were found to migrate together in the 0.58–0.69 and 0.88–1.04 MDa regions, which corresponds to the known migration pattern of MICOS (0.5–1.1 MDa^40^). In *H-Lrpprc*^*−/−*^ mitochondria, the abundance of MICOS subunits, including the CL-binding subunits APOO and APOOL^41^, was generally increased compared to WT.

Prohibitins were next examined as they are known to form large protein complexes that act as lipid scaffolds to regulate CL metabolism and other functions that are crucial for IMM homeostasis^42,43^. As previously shown, the main prohibitin complex formed by PHB1 and PHB2 was detected in the 0.88–1.04 MDa region (Fig. 5c)^44^ together with the 29 S mitoribosomes and other mitoribosome-associated proteins (see Fig. 2e). In *H-Lprrpc*^*−/−*^ mitochondria, the abundance of this complex was increased by 2 folds (Fig. 5c). In addition, multiple other prohibitins-containing complexes running at 1.7 MDa and above were drastically increased in *H-Lprrpc*^*−/−*^ mitochondria compared to WT (Fig. 5c). Several proteases, previously shown to associate with prohibitins to form proteolytic hubs (*i.e*. AFG3L2, SPG7, and YME1L1) were detected in these bands and they were generally more abundant in *H-Lprrpc*^*−/−*^ livers (see confirmatory blots in Fig. S1). Notably, prohibitins complexes above 2.0 MDa co-migrated with Stomatin-Like protein 2 (Stoml2), a member of the stomatin/prohibitin/flotillin/HflK/C (SPFH) family previously shown to interact with prohibitins^43,45^, and to bind CLs to form CL-enriched microdomains for optimal assembly of the ETC complexes^45^. The abundance of Stoml2 in these putative prohibitin complexes was increased by 2.7, 3.5, and 3.7 folds respectively compared to WT.

Since contact sites between the Endoplasmic Reticulum (ER) and mitochondria play an important role in the transfer of lipid precursors required for mitochondrial CL synthesis^30^ we finally looked for potential changes in proteins complexes involved in the maintenance of ER-mitochondria contact sites. In mammalian cells, maintenance of contacts sites is suggested to involve the Endoplasmic Reticulum Membrane Protein Complex (EMC), Mitofusin2 (MFN2) tethers, and Voltage-Dependent Anion Channels (VDACs) - Glucose-Related Protein 75 (GRP75) complexes which are structurally linked to Inositol triphosphate Receptors (IP3R) at the ER surface^46–48^. Although EMC and MFN2 complexes were not captured in our proteomic profiling, VDAC1-3 and GRP75 were detected together in multiple bands, with a main peak in the 0.88–1.04 MDa region, suggesting these proteins migrated as part of a complex (Fig. 5d). In *H-Lprrpc*^*−/−*^ mitochondria, the abundance of VDAC and GRP75 in the 0.88–1.04 MDa region was significantly increased compared to controls (VDACs: 2.7–3.6 fold; GRP75: 1.5 fold vs. *H-Lrpprc*^*+/+*^), as it was in mitochondrial lysates probed with anti-VDAC and GRP75 antibodies (Fig. S5). Furthermore, two additional VDAC-GRP75 containing complexes, which were absent in WT, were observed in the 0.58–0.69 MDa and 2.05–2.45 MDa regions, with a small amount of IP3R being present in the largest of these complexes. The increased abundance of VDAC-GRP75 complexes and their preserved association with IP3R in purified mitochondrial extracts suggest increased ER-mitochondria interactions in *H-Lprrpc*^*−/−*^ mitochondria.

## Discussion

Mouse models of genetic mitochondrial disorders are generally used to understand specific molecular defects and their biochemical consequences, but rarely to map in vivo compensatory mechanisms that allow maintenance of organ function and survival. Here we took advantage of the extraordinary mitochondrial resilience of hepatic *Lrpprc* knockout mice to explore this question using native proteomics and lipidomics. Our data reveal the presence of adaptive changes at multiple levels along the OXPHOS assembly line which likely allow compensating the LRPPRC-dependent mitochondrial translation defect in the liver. We show that beyond stimulation of mitochondrial biogenesis, the abundance of mitoribosomes per unit of mitochondria is increased, and proteostatic mechanisms are stimulated. Furthermore, our results provide novel evidence for stabilization of residual CIV in supercomplexes through a mechanism that may involve changes in the GPL composition of mitochondrial membranes, most notably CLs, which are reported to play a role in stabilizing protein-protein interactions in the inner membrane.

Compensatory upregulation of mitochondrial content in response to LRPPRC deficiency is robust in the liver as shown by TEM imaging and several molecular markers of mitochondrial biogenesis. A similar response is also observed in the heart of cardiac-specific *Lrpprc* knockout mice^6^. Interestingly however, results from a recent study report a strikingly different phenotype following inactivation of *Lrpprc* in mouse brown adipose tissue (BAT), where lack of LRPPRC was associated with a downregulation of mitochondrial biogenesis signaling, a reduced expression of multiple nuclear-encoded oxidative and thermogenic genes, and an accumulation of intracellular triglycerides^49^. At least for mitochondrial biogenesis, there is thus a clear tissue-specificity in the response to the LRPPRC-dependent translation defect, which is likely attributable to differences in energy requirements across cell types and tissues. In BAT, metabolic reprogramming away from fuel oxidation and toward energy storage most likely represents the optimal compensatory response, while in energy-demanding cells such as hepatocytes, which obligatorily rely of oxidative metabolism, upregulation of mitochondrial content to maintain oxidative capacity is probably crucial to support function and survival.

In this regard, is it interesting to note that in patients with mitochondrial diseases, massive mitochondrial proliferation is typically observed in muscle fibers^15,16^, which also supports the notion that compensatory mitochondrial biogenesis occurs preferentially in cells/tissues with high metabolic demands. Intriguingly, this mitochondrial proliferation phenotype is almost invariably observed in muscle fibers from patients harboring mtDNA mutations that affect global translation and result in multi-complex deficiencies, while it is always absent when mutations affect specific OXPHOS subunits^15,16^. Based on the above mentioned dichotomic response of mitochondrial biogenesis to LRPPRC deficiency in liver and myocytes *vs*. BAT^49^, the presence of compensatory mitochondrial biogenesis in patient muscle would therefore appear to be linked to the severity of the OXPHOS impairment as opposed to being specific to translation defects per se.

LRPPRC, in complex with its binding partner SLIRP, was recently shown to bind throughout the mitochondrial transcriptome (with a preference for mRNA) where it acts as a global RNA chaperone that stabilizes RNA structures, allowing exposure of the required sites for translation, stabilization, and polyadenylation of mitochondrial transcripts^17^. Therefore, although CIV is the main OXPHOS complex biochemically impaired in the liver^6,11,14^, lack of LRPPRC clearly affects the secondary structure and stability of the entire mitochondrial transcriptome^10,14,17^. The present results provide evidence that in vivo this is compensated quantitatively by an increase in the number of mitoribosomes per mitochondrial unit, which was also reported in the cardiac-specific *Lrpprc* knockout model^6^. This phenomenon is likely important for preserving a balance in the availability of mitochondrial- *vs* nuclear-encoded OXPHOS complex subunits in presence of a strong LRPPRC deficit, particularly in the face of a greater drive for mitochondrial biogenesis which increases the supply of nuclear-encoded polypeptides.

Over the last decade, studies have uncovered the mtUPR, a transcriptional program activated by mitochondrial proteotoxic stress, which aims to maintain proteostasis within the organelle. Studies in yeast and mammalian cell models have shown that this program triggers the genetic activation of several mitochondrial chaperones and proteases^19,50,51^. The classic mtUPR also causes a reduction in the availability of mitochondrial transcripts, potentially through transcriptional repression at the level of the D-loop on mtDNA, and reduced expression of nuclear-encoded OXPHOS genes^19^. Furthermore, the mtUPR is reported to trigger broader changes such as the suppression of general translation and privileged translation of genes involved in cell survival, which in mammalian cells appears to be linked to the Integrated Stress Response pathway^19^. However, limited data is available on the mtUPR response in the liver of in vivo models of genetic mitochondrial diseases. Our results show that LRPPRC deficiency in the liver potently induces the expression of CHOP, a key transcriptional activator of the mtUPR and ER stress responses, and increases the steady-state levels of several chaperones involved in mitochondrial proteostasis. However, this proteostatic response occurs without signs of reduced translation of nuclear and mitochondria-encoded mitochondrial genes, which are hallmarks of the classic mtUPR in yeast and mammalian cell models^19,50^. Thus, in vivo, and in an energy-demanding tissue such as the liver, the mtUPR differs from the classic response, which likely reflects the need to balance proteostasis while maintaining energy homeostasis. Therefore, our results suggest the mtUPR allows to achieve higher levels of chaperone and proteases required to support mitochondrial biogenesis in the context of a mitochondrial translation defect that inevitably perturbs the dynamic equilibrium between steady-state levels of mtDNA- and nuclear-encoded polypeptide.

Beyond quantitative adjustments in mitochondrial biomass and translation capacity, our study suggests that stabilization of CIV into SCs may optimize its residual activity. Stabilization of CIV was apparent both in liver mitochondria from LRPPRC-deficient mice and fibroblastic mitochondria from LSFC patients, where the proportion of CIV^14^ incorporated in the S_1_ respirasome relative to its monomeric form was increased. Although the high degree of conservation of SCs across species logically implies that they confer some functional advantages, the true physiological *raison d’être* of these supramolecular structures still remains unclear^21^. One possibility is that SCs formation increases ETC efficiency by facilitating electron flow between complexes through substrate channeling^51–54^. This mechanism could explain why maximal uncoupled flux through the electron transport chain is completely preserved, and the respiratory rate in presence of CIV substrates (e.g. TMPD/Ascorbate) is only reduced by 20–30% in *H-Lrpprc*^*−/−*^ mitochondria despite a drastic reduction (80–85%) in the amount of assembled CIV (^14,55^and Fig. S2).

The formation of SCs is also suggested to reduce ROS production at the level of CI and/or CIII^56,57^. Interestingly, our previous study showed that superoxide release by the ETC is increased in presence of low LRPPRC levels^14^. In this context, preferential integration of CIV in CI-CIII containing SCs could act as a compensatory mechanism to limit further oxidative damage to mitochondria. Some studies^58,59^, but not all^55^, have also suggested SCs could provide a scaffold for the completion of CI assembly and/or its stabilization. Accordingly, preferential integration of residual CIV in SCs could act to prevent CI abnormalities secondary to the CIV assembly defect. However, considering the severity of the CIV assembly deficit and the complete absence of CI defects observed in LRPPRC deficient mitochondria, this possibility seems unlikely.

Although the existence of SCs as physical entities is now well accepted, the molecular mechanisms regulating their formation remain poorly understood^21^. Previous studies have shown that the CIII-associated protein COX7A2L assists and/or stabilizes the interactions between CIII and CIV, and consequently the formation and/or stability of III_2_+IV_1_ and I/III_2_/IV supercomplexes^22,25,51,60^. In the present study, we found that the expression of COX7A2L was increased in mitochondria from H-*Lrpprc*^*−/−*^ mice, and was stabilized in III_2_ and I_1_-III_2_ assemblies. As suggested recently, this could be part of a response to accelerating CIII-containing SCs assembly under stress conditions^52^. However, when the short variant of COX7A2L is expressed, as in our mouse model, this compensatory mechanism cannot successfully stabilize CIV since the 6 bp deletion prevents interactions with CIV^21,22,25^, as shown by the near absence of COX7A2L in CIV-containing SCs both in WT and *H-Lrpprc* knockout mice. These results therefore provide clear evidence that the preferential integration of CIV is caused by other mechanisms.

Factors such as membrane GPL composition play a role in the assembly and maintenance of SCs^29,30,32^. Early studies in yeast have established an important contribution of CL in stabilizing respiratory chain SCs^32,35^. Structural studies employing cryo-electron microscopy have estimated that 50 and 200 CL were present in yeast and bovine SCs respectively^61,62^. Self-assembly molecular dynamics simulations performed in bovine heart complexes embedded in CL-containing bilayers, which aimed to mimic the crowdedness and complexity of mitochondrial membranes, have also revealed that CL enrichment at the SC interface precedes their assembly and act as a strong glue acting at specific sites on the surface of proteins^33^. Based on these studies, our results suggest that the upregulation of CLs may contribute to the stabilization of residual CIV in SCs. This stabilizing role of CLs is further suggested by our observation that in native gel studies, several protein complexes, known to require CLs for their assembly and/or stability, are more abundant in *H-Lrpprc*^*−/−*^ mitochondria. This includes a minor fraction of the mitochondrial ANT which is able to associate with SCs in a strict CL-dependent manner^37^, as well as MICOS and Prohibitin/Stoml2 complexes which, apart from being stabilized by CLs, also play a role in CL synthesis, and the formation of CL-rich microdomains in the inner mitochondrial membrane^38,39,42,43^. Prohibitin/Stoml2 complexes in particular were suggested to form CL-enriched microdomains in which electron transport complexes are optimally assembled^45^.

Our native gel proteomics data also revealed that the abundance of GRP75-VDAC-IP_3_R complexes is increased in LRPPRC deficient mitochondria, which raises the intriguing possibility of an increased tethering between mitochondria and ER^46,47^. By generating closely appositioned membranes, these tethers are increasingly viewed as being important for the movement of GPL across cellular compartments^30^. In yeast for instance, impairment of ER-mitochondria tethering was recently shown to decrease the transfer of PA from ER to mitochondria, resulting in a 40% reduction in CL levels^63^. Thus, increased tethering between mitochondria and ER could promote GPL trafficking between ER and mitochondria and at least partly account for the increased abundance of CLs. The fact that nearly all of the differentially abundant PEs detected in our lipidomics analysis were upregulated in LRPPRC-deficient mitochondria is also suggestive of altered phospholipid trafficking between ER and mitochondria, as the ER and ER-associated membranes (MAMs) play a key role in mitochondrial PE synthesis^64^.

Although indicative, these results only constitute indirect evidence. Further studies in which upregulation of CLs is prevented in liver-specific *Lrpprc*-deficient mice will be required to confirm that changes in the lipid milieu underlie the stabilization of CIV. Interestingly, recent evidence indicates that greater mitochondrial PE could also potentiate respiratory enzymes activity^65^. Our lipidomic analysis pointing to a relative increase of PE species, compared to PC species, supports the notion that GPL remodeling may be a mechanism to preserve or potentiate the activity of the residual CIV in H-*Lrpprc*^*−/−*^ mice.

## Methods

### Animal care

All experiments on animals were approved by the University of Ottawa Institutional Animal Care Committee and conducted according to the directives of the Canadian Council on Animal Care. Mice were maintained in ventilated cage racks by groups of 5 mice. All mice of both sexes were kept on a regular 12–12 h light-dark cycle, and had access to food and water *ad libitum*. Animals were used at 5 weeks of age for experiments and euthanized by cervical dislocation.

To disrupt LRPPRC expression in the liver, the *Lrpprc* knock-out mouse line *Lrpprc*^*tm1a(KOMP)Wtsi*^ produced in C57BL/6 N embryonic stem (ES) cells were acquired from the KOMP Repository (University of California, California) as previously reported^14^. The mutated locus was transmitted through the germline to obtain heterozygous *Lrpprc*^*+/lox-neo*^ animals. These animals were then bred with *flp* producing animal B6(C3)-Tg(Pgk1-FLPo)10Sykr/J (The Jackson Laboratory) in order to excise the neomycin resistance cassette, recreating an LRPPRC protein-producing locus having *lox* sites in intron 3 and 5. The resulting *Lrpprc*^*+/loxP*^ mice were then mated to get homozygous *Lrpprc*^*loxP/loxP*^ mice and only animals that were exempt of the *flp* allele were kept. To achieve liver-specific inactivation of Lrpprc, *Lrpprc* ^*loxP/loxP*^ mice were crossed with B6.Cg-Tg(Alb-cre)21Mgn/J (The Jackson Laboratory) mice producing the Cre recombinase under the control of the albumin promoter and bred to homozygous state, Hep-Cre^cre/cre^. These mice were then bred with the *Lrpprc*^*loxP/loxP*^ to generate double homozygous mice *Hep-Lrpprc*^*loxP/loxP;cre/cre*^. However, in order to simultaneously generate homozygous knockout and wild-type littermate controls, Hep-*Lrpprc* ^*loxP/loxP;cre/0*^ individuals were inter-crossed.

### Transmission electron microscopy

Following anaesthesia (8% chloral hydrate; 600 mg/kg), mice were perfused with 10 ml PBS and 5 ml 2,5% glutaraldehyde via the vena cava. Livers were excised, sliced, and fixed overnight at 4 °C in 2,5% glutaraldehyde in phosphate buffer. After sample preparation, 90–100 nm thick sections were mounted onto a 200 mesh copper grid (Electron Microscopy Sciences) and imaged with an FEI Tecnai 12120 kV transmission electron microscope equipped with an AMT XR80C 8 megapixel CCD camera as previously described^66^.

### Mitochondria isolation

Hepatic mitochondria were isolated from 5 weeks old mice by differential centrifugation as described previously^14^. Isolated mitochondria were either kept on ice and immediately used to measure mitochondrial respiration or stored at −80 °C until SCs sample preparation.

For fibroblasts mitochondria, confluent cells (total of 30–60 × 106 for one SCs extraction sample) were lifted using trypsin, washed with PBS and the cell pellet was flash frozen and kept at −80 °C. After thawing, the cell pellet was resuspended in 500ul buffer A (220 mM Mannitol, 20 mM HEPES, 70 mM Sucrose, 1 mM EDTA, pH7,4, 1× protease inhibitor, 2 mg/ml BSA), transferred to a glass-glass homogenizer, and volume was completed to 5 ml with buffer A. Cells were broken with 15 strokes with the loose pestle followed by 30 strokes with the tight pestle. Cell breakage was assessed using a light microscope. Homogenate was transferred to a Nalgene centrifuge tube, volume was completed to 30 mL, and centrifuged at 700 g for 5 min at 4 °C. The supernatant was transferred in a new tube and centrifuged at 10,000 g for 10 min at 4 °C. Pellet was resuspended in 2 mL buffer B (220 mM Mannitol, 20 mM HEPES, 70 mM Sucrose, 1 mM EDTA, pH7,4, 1× protease inhibitor), volume completed to 30 mL, and centrifuged at 10,000 g for 10 min at 4 °C. The washing step was repeated a second time and the final mitochondria pellet was resuspended in 1 mL buffer B. From this point, fibroblasts mitochondria were treated like the liver mitochondria for SCs extraction, using a ratio of 5 g digitonin/1 g of mitochondria protein for the extraction.

### mtDNA copy

mtDNA isolation and copy number analysis were performed as previously described^67^. Liver tissue (30 mg) from 5 weeks old animals were digested in proteinase K (0.2 mg/ml) for 3 h at 55 °C. The lysate was subjected to RNase A (100 μg/ml) for 30 min at 37 °C. DNA was pelleted following the addition of ammonium acetate-isopropanol (7.5 M; 0.7 v/v) and centrifugation at 15,000 g for 10 mins at 4 °C. Pellet was washed with 70% ethanol and resuspended in TE buffer. DNA (20 ng) was combined with SsoAdvanced Universal SYBR Green Supermix (Bio-Rad), with ND1(mtDNA) or HK2 (nuclear DNA) primer sets (ND1 FWD: 5′-CTAGCAGAAACAAACCGGGC-3′; REV: 5′-CCGGCTGCGTATTCTACGTT-3′; HK2: FWD: 5′-GCCAGCCTCTCCTGATTTTAGTGT-3′; REV: 5′-GGGAACACAAAAGACCTCTTCTGG-3′). Plate (96 well) was read using CFX Real-Time PCR (Bio-Rad; 95 °C for 5 min, 45 cycles of 95 °C for 10 s, 60 °C for 10 s and 72 °C for 20 s). The ΔCt of ND1/HK2 was used to calculate the copy number using the equation 2ΔCt.

### Electrophoresis

#### Western blotting

Whole livers were homogenized in RIPA buffer, centrifuged and the supernatant was collected, proteins were quantified using Pierce BCA protein assay kit (Thermo Scientific). Extracts were mixed in a 1:1 ratio with laemlli buffer before being loaded on polyacrylamide gels (40 μg protein per well, 7.5–15% gels depending on protein of interest). Gels were submitted to a 1 h 30 run at 150 V, then retrieved and transferred to PVDF membranes at 110 V for 1 h at 4 °C. Membranes were blocked in 5% milk in TBST and incubated in antibody solution (1:1000 dilution) O/N at 4 °C. After incubation in secondary antibody solution for 1H, membranes were imaged with Chemidoc (Bio-Rad) using enhanced chemiluminescence (Amersham ECL prime detection reagent, GE). Protein abundance was normalized to Ponceau staining of membranes after antibody detection.

#### CN/BN-PAGE, 2D-SDS-PAGE, 2D-DDM-CN/BN-PAGE

Briefly, SCs were extracted from liver mitochondria using digitonin (4 g digitonin/g of mitochondrial protein) solubilized in an extraction buffer. SCs were resolved in large native gradient gels (4–12%) with 175 µg of proteins in each well as previously described^68^. For patient fibroblasts mitochondria, a digitonin ratio of 5 g/1 g was used, and 100 µg of protein were loaded in each well of a 4–12% gradient native gel. For hybrid CN/BN-PAGE, the first hour and a half of electrophoresis were performed in presence of 0.022% Coomasie Blue in cathode buffer, and the remaining without^68^. After O/N electrophoresis at 4 °C, gels were retrieved for either in-gel activity assays or transferred to PVDF membranes for antibody detection. Antibodies used at a 1:1000 dilution include: NDUFA9 (CI, Invitrogen, #459100); SDHA (CII, Abcam, #ab14715); UQCRC2 (CIII, Abcam, #ab14745); COX4 (CIV, Invitrogen, #459600), ATPB (CV, Abcam, #ab14730), and COX7A2L (Abcam #ab66107).

### Proteomics profiling

For proteomics profiling, replicate samples from 6 mice (3 *H-Lrpprc*^*−/−*^ and 3 *H-Lrpprc*^*+/+*^) were resolved by Hybrid CN/BN PAGE. In-gel activity assays for OXPHOS complexes were performed and the staining pattern obtained was aligned and used as a guide to cut bands in the unstained replicate that was migrated in parallel. For each sample, a total of seven bands of equal size (5 mm height) were cut covering the following molecular weight ranges (in MD): 2.9 ± 0.253, 2.6 ± 0.22, 2.25 ± 0.20, 1.85 ± 0.16, 1.55 ± 0.14, 1.0 ± 0.083, and 0.65 ± 0.05. Proteins were digested in-gel using trypsin (Promega) according to the method of Shevchenko^69^. Peptide extracts were then concentrated by Vacufuge (Eppendorf), and diluted to 20 µl in 0.1% formic acid/water.

5% of each digest was injected and analyzed by liquid chromatography-tandem mass spectrometry (LC-MS/MS) in an Orbitrap Fusion Lumos Mass Spectrometry System (Thermo Scientific) with a Dionex Ultimate 3000 RSLC Nano High-Performance Liquid Chromatography system (Thermo Scientific) at the front end and the Xcalibur 4.0.27.10 software package. Peptides were separated on a C18 PepMap100 precolumn, and a C18 2 µ, Acclaim PepMap RSLC column (Thermo Scientific) using a 15 min gradient of 5 to 36% acetonitrile with 0.1% formic acid at a flow rate of 300 nL/min. Peptides were analyzed using Data Dependent Acquisition (DDA) mode. Full scan MS mode (400–1500 m/z) was operated at a resolution of 120,000 with automatic gain control (AGC) target of 4 × 10^5^ ions, and a maximum ion transfer of 35 ms. Selected ions for MS/MS were analyzed using the following parameters: resolution 120,000; AGC target of 4 × 10^5^; maximum ion transfer of 35 ms; 1.6 m/z isolation window; for CID normalized collision energy of 35% was used; and dynamic exclusion of 60.0 s. The Easy-IC was used for internal calibration.

### Protein identification and data analysis

The analysis of the RAW files was performed using the MaxQuant software package (version 1.6.5.0). The extracted spectra were matched against the mouse sequences from SwissProt (version 2019–04). Sequences of known contaminants were added to this database, and the reverse decoy was strictly set to FDR of 0.01. Database searches were done with 20 ppm and 0.5 Da mass tolerances for precursor ions and fragmented ions respectively. Trypsin (two missed cleavages allowed) was selected as the protease. Dynamic modifications included N-terminal acetylation and oxidation of methionine, while Cystein carbamidomethylation was set as a fixed modification.

Protein abundances were determined by label-free quantitation using the composite iBAQ intensity values determined by MaxQuant and normalized within single or multiple migration profiles of individual proteins. Normalized protein quantification values were then imported into the Perseus^70^ software platform for log transformation, data imputation, and statistical analysis. For proteins missing values for 1 of the 3 experimental replicates in a particular band and genotype, the mean iBAQ value of the two other replicates was imputed. All proteins missing more than one value in a particular band and genotype were considered undetected. For each protein, gel migration profiles were created and normalized to the maximum abundance across all samples analyzed. The migration profile of proteins belonging to known multi-protein complexes were then manually extracted and heatmaps of relative abundance were generated using GraphPad Prism 8 software package (version 8.4.2) to visualize co-migration. The absolute abundance of selected protein complexes was also estimated by summing the iBAQ values of all detected protein components co-migrating in the same band. For statistical analysis, one sample *T* tests comparing the two genotypes were performed for each band. A *P* value of less than 0.05 was set as the arbitrary threshold for statistical significance. For each *P* value obtained, a corresponding False Discovery Rate (FDR) was calculated according to the Benjamini and Hochberg method and lists of significantly affected proteins were generated at FDR thresholds of < 5, < 10, and < 15% FDR values. The mass spectrometry proteomics data have been deposited to the ProteomeXchange Consortium via the PRIDE^71^ partner repository with the dataset identifier PXD021867.

### Quantitative RT-PCR

Chop transcript level from frozen liver tissue was assessed using an RNeasy kit (QIAGEN) for RNA extraction and reverse transcribed with the High-Capacity CDNA RT kit (Thermo Fisher Scientific) according to the manufacturer’s recommendations. Quantification was allowed using the ∆∆CT method and the TATA Binding Protein gene expression was used as a housekeeping gene.

*Primers used: Chop (NM_007837), sense 5*′*-TATCTCATCCCCAGGAAACG-3*′*, anti-sense 5*′*-CAGGGTCAAGAGTAGTGAAGGTTT-3*′*; Tbp (NM_013684), sense 5*′*-GGCCTCTCAGAAGCATCACTA-3*′*, anti-sense 5*′*-GCCAAGCCCTGAGCATAA-3*′.

### Lipidomics

Lipid extraction and data analysis were done as previously described^31^ and adapted for isolated mitochondria^14^. In brief, lipids were extracted from isolated mitochondria or digitonin-extract and spiked with six internal standards: LPC 13:0, PC19:0/19:0, PC14:0/14:0, PS12:0/12:0, PG15:0/15:0, and PE17:0/17:0 (Avanti Polar Lipids Inc, Alabaster, USA). For both kinds of samples, protein concentration was determined using a colorimetric-based assay based on the Bradford dye-binding method to set the volume of injection. Samples were injected (isolated mitochondria: from 1.03–4 μl equivalent to 25 µg of protein extract; digitonin-extract: from 0.98–1.20 μl equivalent to 1.5 µg of protein extract) into a 1290 Infinity HPLC coupled with a 6530 Accurate Mass Q-TOF (Agilent Technologies Inc., Santa Clara, USA) via a dual electrospray ionization (ESI) source. Elution of lipids was assessed on a Zorbax Eclipse plus column (C18, 2.1 × 100 mm, 1.8 μm, Agilent Technologies Inc.) maintained at 40 °C using an 83 min chromatographic gradient of solvent A (0.2% formic acid and 10 mM ammonium formate in water) and B (0.2% formic acid and 5 mM ammonium formate in methanol/acetonitrile/methyl tert-butyl ether [MTBE], 55:35:10 [v/v/v]). The analysis was performed in positive scan mode. MS data processing was achieved as previously describe^31^ using Mass Hunter B.06.00 (Agilent Technologies Inc.) and a bioinformatics pipeline that we have developed to perform several steps including: (i) ensuring optimal MS alignment between chromatographic runs, (ii) filtering of adducts as well as subsequent data mining done using an in-house script that applies frequency filtering (80% in one condition), (iii) signal intensity normalization using cyclic loess algorithm and (iv) imputation of missing values with a k-nearest neighbor on scaled data. This yields a data set listing MS features characterized by mass, retention time, and corrected signal intensity. Lipid annotation, including fatty acids side chains, was achieved by MS/MS on all discriminant MS features selected according to the following criteria of selection: *Q*-value < 0.05 (as an estimation of false discovery rate (FDR) < 5%) and a relative fold change (FC) of 1.5 or 0.75. Statistical analysis was performed on the data using an unpaired student’s *t*-test with Storey correction for multiple testing using the *Q*-value package from Bioconductor. Data are depicted as volcano plots and box plots, where the midline represents the median fold change vs. controls, the box represents the interquartile range (IQR) between the first and third quartile, and whiskers represent the lowest or highest values.

### Statistics and Reproducibility

For functional immunoblot and Q-PCR experiments, values are reported as mean ± sem for a minimum of 3 mice per experimental group and graphically represented as violin plots. Unpaired two-sided *t*-tests with Welch’s correction (GraphPad Prism 8.4.3) were used to determine statistical difference when two means were compared, with a significance threshold set at *p* < 0.05.

For lipidomics, data are depicted as volcano plots and box plots, where the midline represents the median fold change vs. controls, the box represents the interquartile range (IQR) between the first and third quartile, and whiskers represent the lowest or highest values (*n* = 4 mice per group). All discriminant MS features were selected according to the following criteria of selection: *Q*-value < 0.05 (as an estimation of false discovery rate (FDR) < 5%) and a relative fold change (FC) of 1.5 or 0.75. Statistical analysis was performed on the data using an unpaired student’s *t*-test with Storey correction for multiple testing using the *Q*-value package from Bioconductor.

For proteomics, replicate samples from 6 mice (3 *H-Lrpprc*^*−/−*^ and 3 *H-Lrpprc*^*+/+*^) were analyzed. The difference in the abundance of individual proteins between genotypes was determined in each band using unpaired two-tailed *T* tests. A *p* value of less than 0.05 was set as the arbitrary threshold for statistical significance. For each *p* value obtained, a corresponding FDR was calculated according to the Benjamini and Hochberg method, and lists of significantly affected proteins were generated at FDR thresholds of < 5, < 10, and < 15% FDR values (Supplemental Table 1). To test differences in the absolute abundance of selected multi-protein complexes across genotypes, unpaired two-sided *t*-tests were performed. p values were corrected using the Benjamini & Hochberg method with an FDR cutoff of 5%. Corrected *p* values (*Q*)< 0.05 were considered significant.

### Reporting summary

Further information on research design is available in the Nature Research Reporting Summary linked to this article.

## Supplementary information

Description of Additional Supplementary Files

Supplementary Information

Supplementary Data 1

Supplementary Data 2

Reporting Summary

## Data Availability

The mass spectrometry proteomics data have been deposited to the ProteomeXchange Consortium via the PRIDE ^79^ partner repository with the dataset identifier PXD021867. Source data used to generate figures are provided as supplemental data [Supplementary Data 2]. All other data are available from the corresponding authors upon reasonable request.
