# Supplementary material for: Adaptive optimization of the OXPHOS assembly line partially compensates lrpprc-dependent mitochondrial translation defects in mice
Source: Commun Biol. 2021 Aug 19;4:989. doi: 10.1038/s42003-021-02492-5 (PMC8376967; doi:10.1038/s42003-021-02492-5)
Supplement: Supplementary file 1 — Description of Additional Supplementary Files [file 42003_2021_2492_MOESM1_ESM.pdf]

Description of Additional Supplementary Files

File name: Supplementary Data 1

Description: List of identified proteins by complexome profiling.

File name: Supplementary Data 2

Description: Source data used for figures.
