# Supplementary material for: Adaptive optimization of the OXPHOS assembly line partially compensates lrpprc-dependent mitochondrial translation defects in mice
Source: Commun Biol. 2021 Aug 19;4:989. doi: 10.1038/s42003-021-02492-5 (PMC8376967; doi:10.1038/s42003-021-02492-5)
Supplement: Supplementary file 2 — Supplementary Information [file 42003_2021_2492_MOESM2_ESM.pdf]

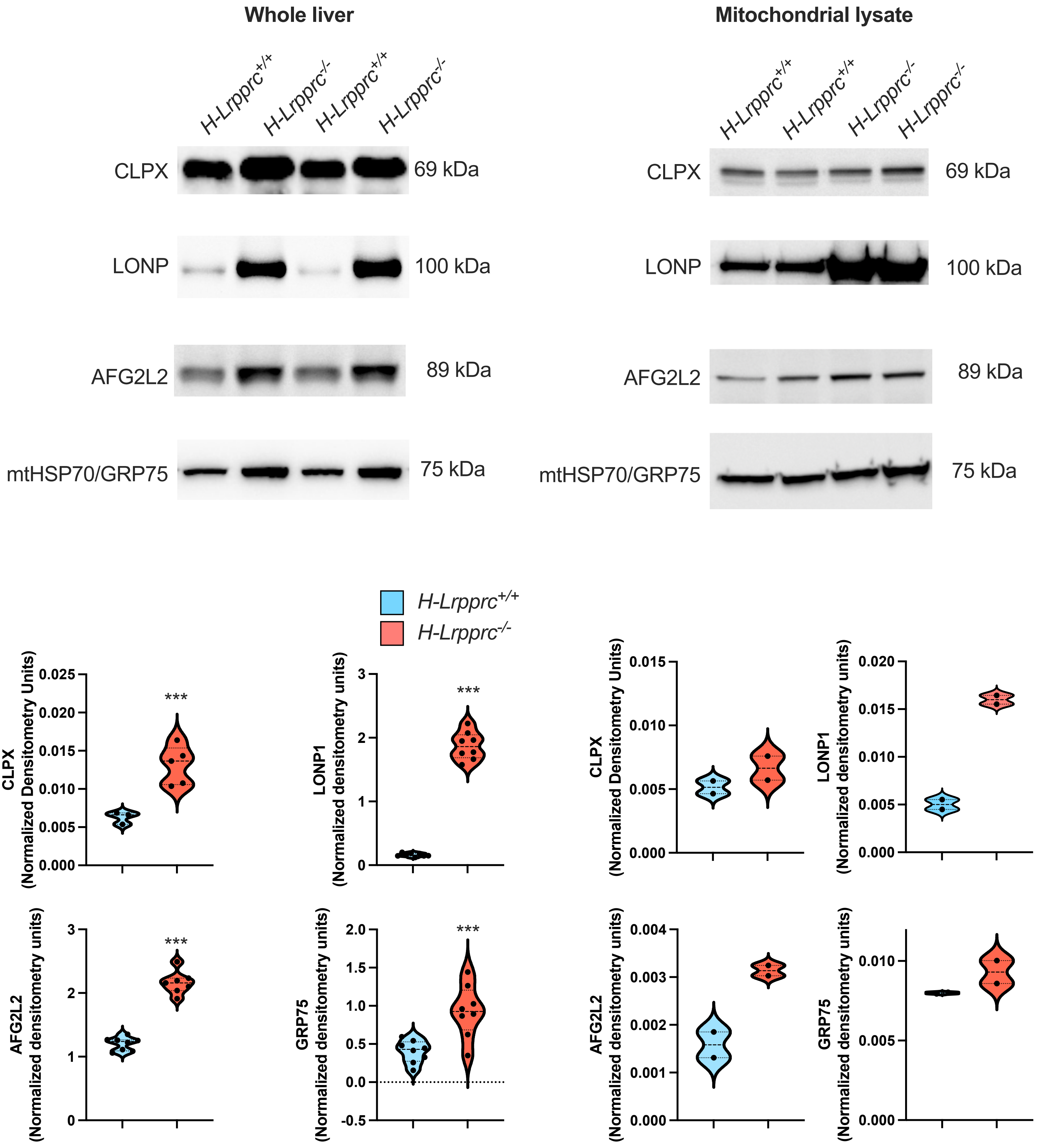

Fig S1: Impact of LRPPRC deficiency on protein abundance of selected proteases. Figure shows immunoblots performed following SDS-PAGE of liver lysates and mitochondrial lysates. Bar graphs represent mean ± sem of the quantitative analysis of protein expression performed on 5-8 animals per experimental groups. Densitometry values obtained for each protein was normalized against the Ponceau stain (provided in Fig S6). Unpaired two-sided T tests with Welsh's correction was used to assess significance (**: $P<0.01$).

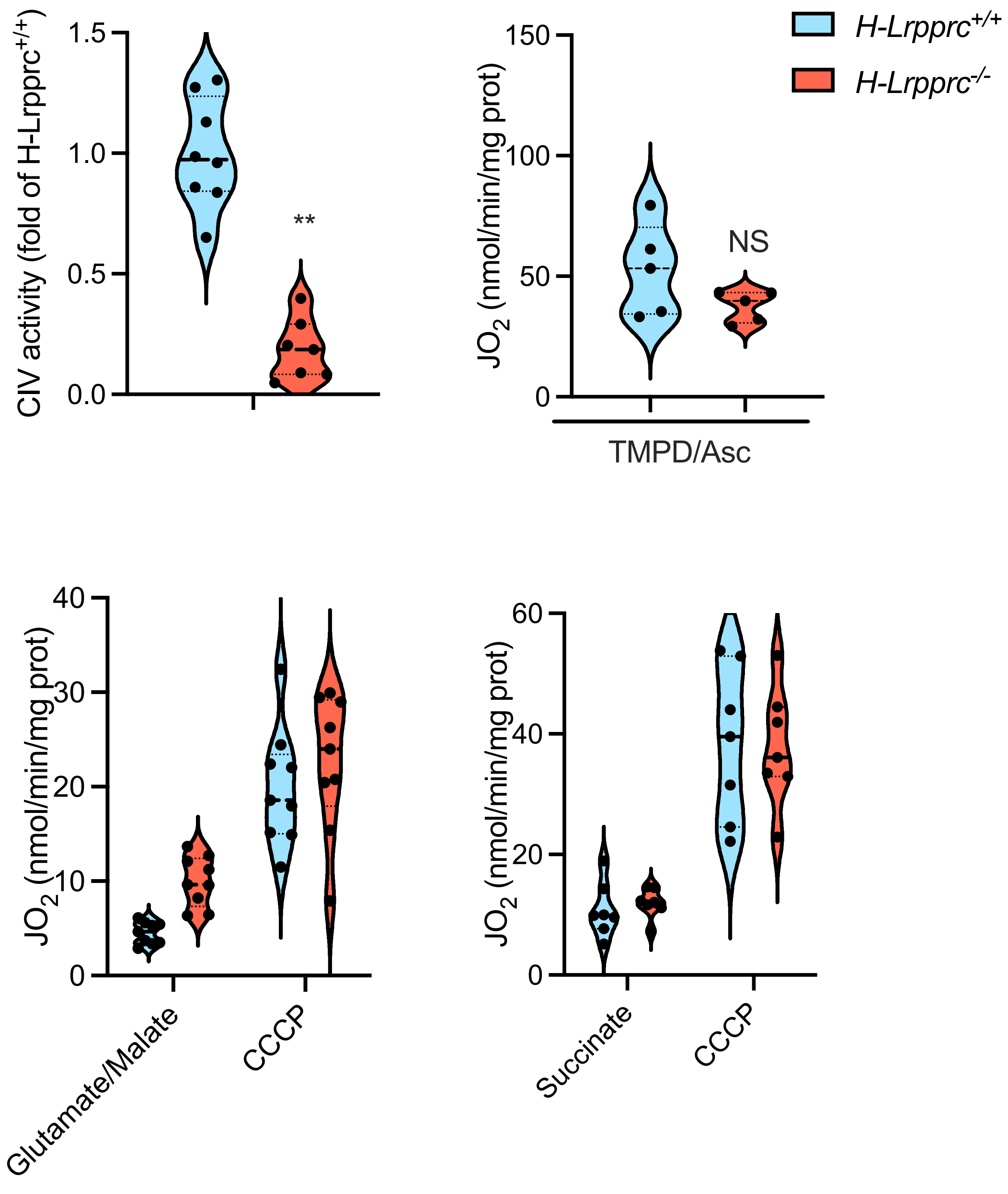

Fig S2: Impact of LRPPRC deficiency on CIV activity and respiratory capacity: Panel A: CIV activity measured spectrophotometrically in isolated liver mitochondria following detergent extraction. Panel B: Maximal state 3 respiration in isolated liver mitochondria energized with the CIV substrates TMPD and Ascorbate (9mM/0.9mM). Panel C-D: Baseline and maximal CCCP uncoupled respiration in mitochondria energized with CI (5 mM glutamate-0.5 mM malate) and CII (5 MM succinate) donors. Unpaired two-sided T tests with Welsh's correction was used to assess significance (**P<0.01).

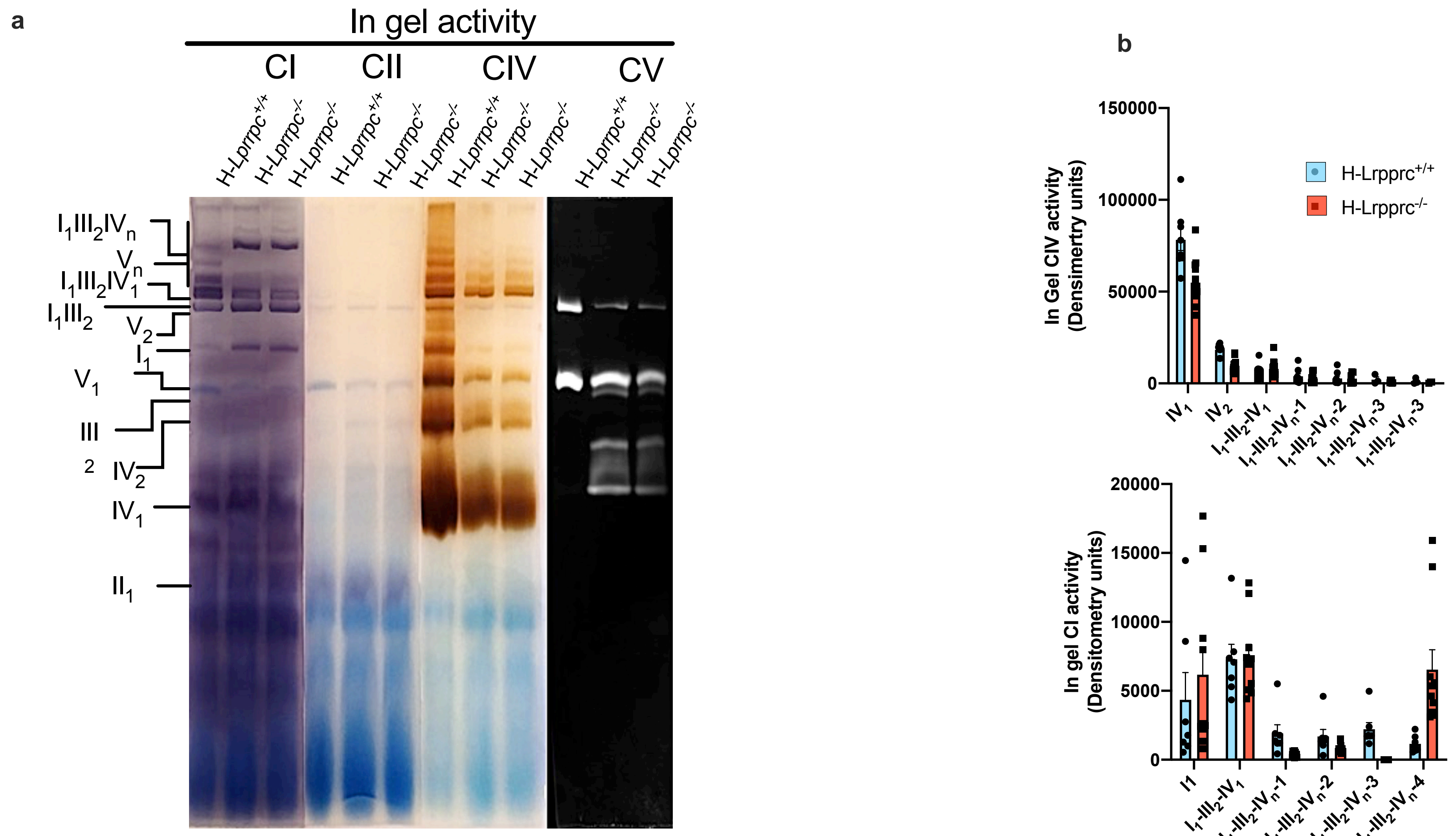

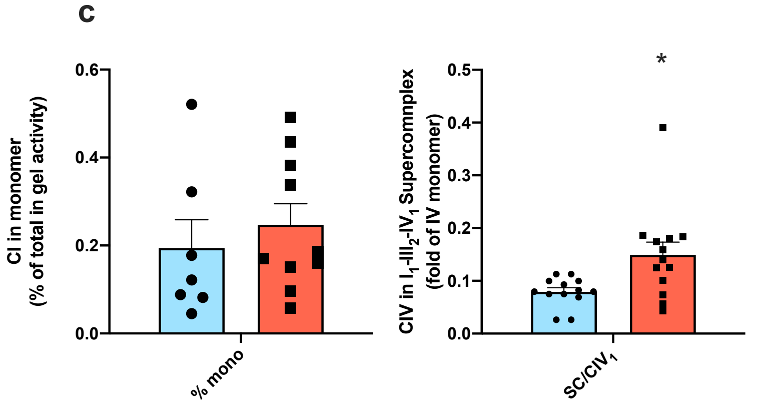

Figure S3: Impact of LRPPRC deficiency on respiratory chain supercomplexes assessed by in-gel activity. Panel A: Representative migration pattern of respiratory chain supercomplexes in digitonin-solubilized mitochondrial extracts from *H-Lrpprc*$^{+/+}$ and *H-Lrpprc*$^{-/-}$ mice resolved by hybrid CN/BN-PAGE. Replicates of the same 3 samples (1 *H-Lrpprc*$^{+/+}$ and 2 different *H-Lrpprc*$^{-/-}$) were loaded in multiple wells and migrated together. After electrophoresis, replicates were cut and incubated in the appropriate activity buffer for CI, CII, CIV and CV. Replicates were imaged (Chemidoc, BioRad) and aligned for SCs identification. OXPHOS complexes and supramolecular assemblies are identified using the standard nomenclature, with numbers in indices indicating the molecular stoichiometry of each OXPHOS complex. Panel B: distribution of CIV and I between monomeric form, dimers and supercomplexes species was assessed by measuring the intensity of each reactive band following in-gel activity for CIV and I (Image J) and compared between groups (n= 3-5 mice per group, 2 technical replicate). Panel C: proportion of monomeric CI was calculated using the intensity value following in-gel activity (Image J). Proportion of CIV incorporated in the respirasome was also calculated using the intensity value of the band following in-gel activity (Image J). Unpaired two-sided T tests with Welsh's correction was used to assess significance (*P<0.05).

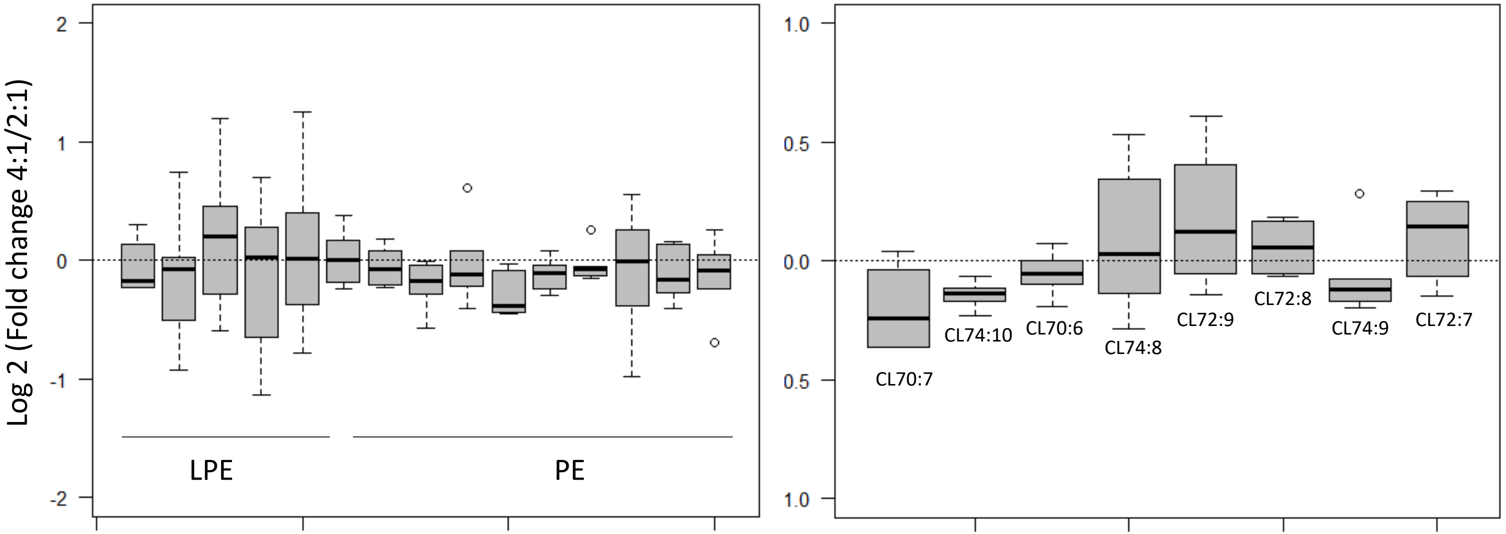

Figure S4: Impact of digitonin concentration on the glycerophospholipid (GPL) profile detected in supercomplex extracts. Figure shows box plots comparing the abundance of selected lysophosphatidyl ethanolamine (LPE), phosphatidylethanolamine (PE) and cardiolipin (CL) in supercomplexes extracted using a 4:1 or a 2:1 digitonin/protein ratio (n= 6-7 per condition). Data are presented as log2 ratio between abundancies measured at 4:1 vs 2:1. Absence of significant deviation from zero indicates that digitonin concentration has a negligible impact on the GPL profile in supercomplex extracts.

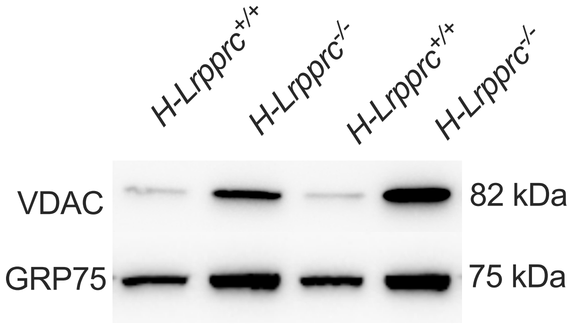

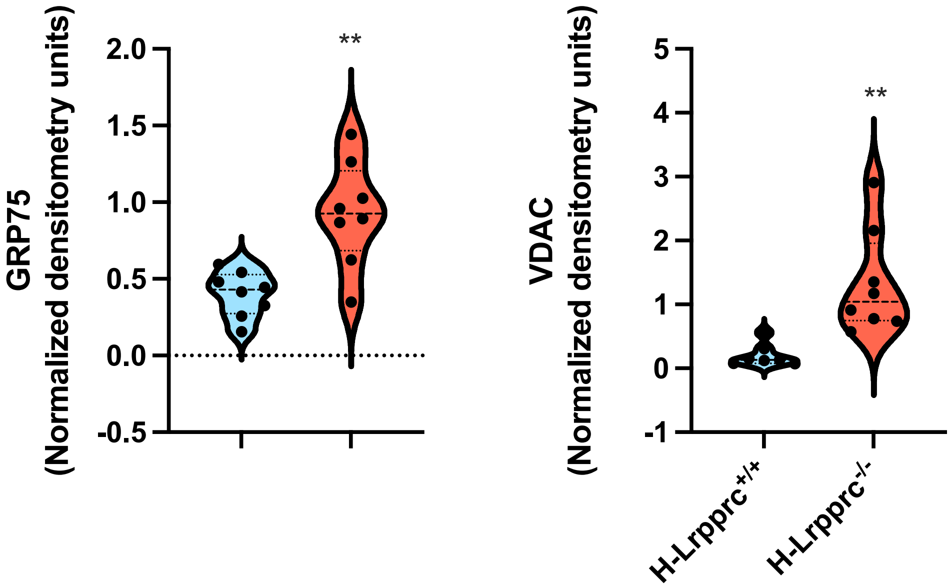

Fig S5: Impact of LRPPRC deficiency on the abundance of ER-mitochondria tethering proteins. Figure shows representative immunoblot performed following SDS-PAGE of whole liver lysates. Bar graphs represent mean ± sem of the quantitative analysis of protein expression performed on 7-8 animals per experimental groups. Densitometry values obtained for each protein was normalized against the Ponceau stain (provided in Fig S6). Unpaired two-sided T tests with Welsh's correction was used to assess significance (**: $P<0.01$).

Figure 1-C: PGC1-α

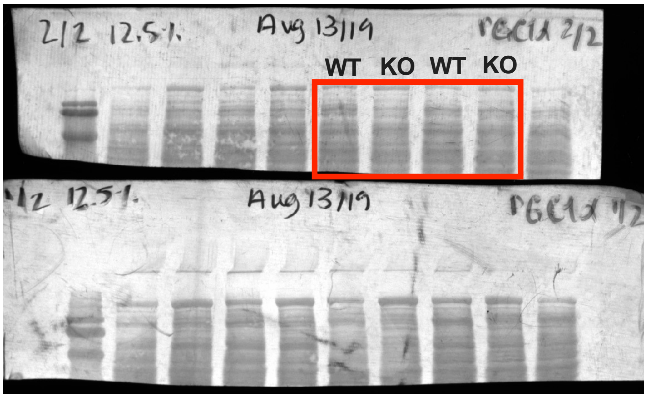

Figure 1-C: Tfam

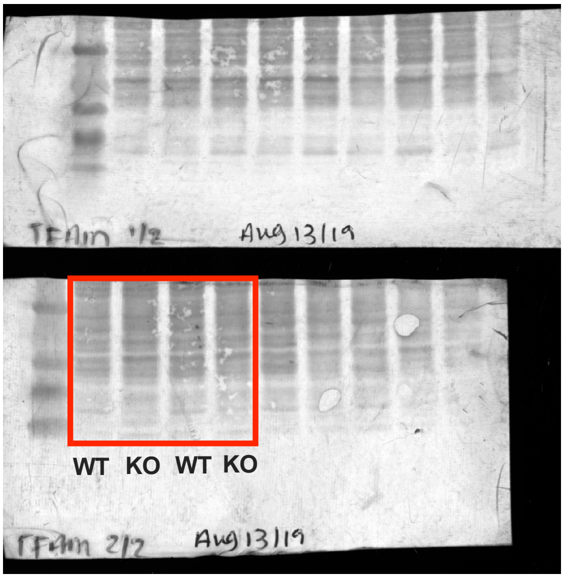

Figure 3-A: COX7A2L

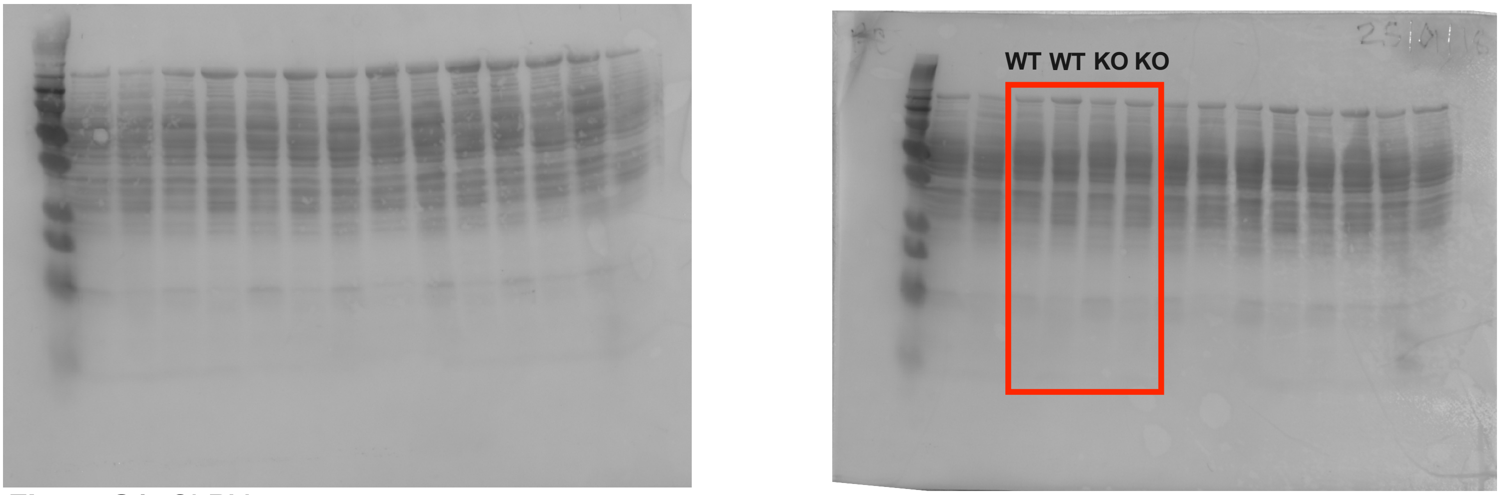

Figure S1: CLPX

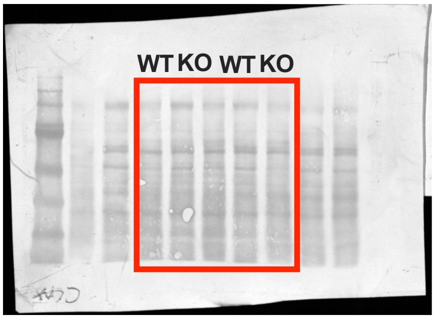

Figure S1: LONP

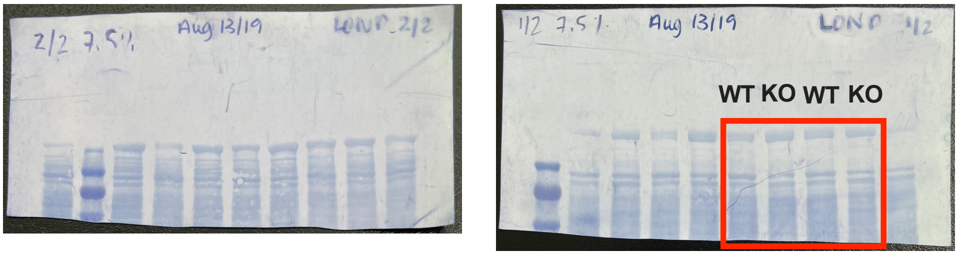

Figure S1: AFG3L2

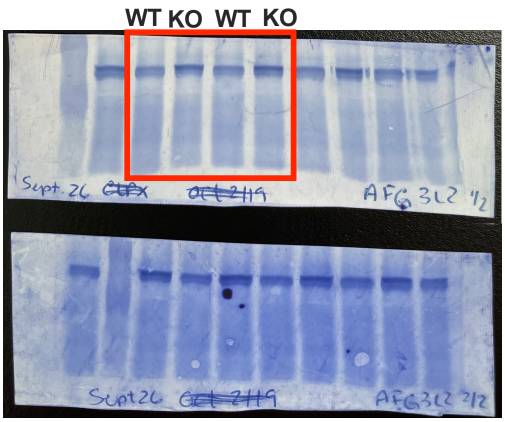

Figure S1: GRP75

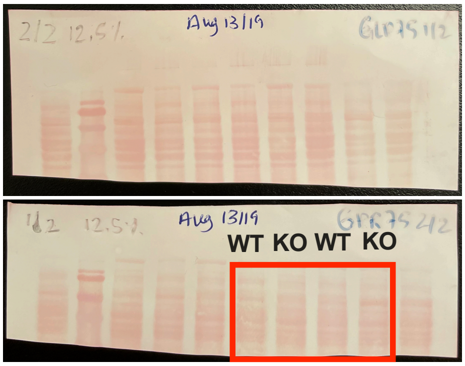

Figure S5: VDAC

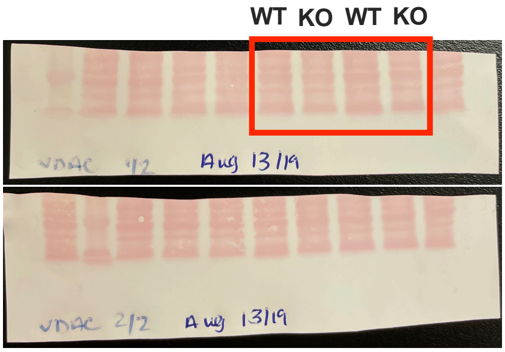

Fig S6: Coomassie blue and Ponceau straining of membranes used for immunoblot experiments reported in the indicated figures.

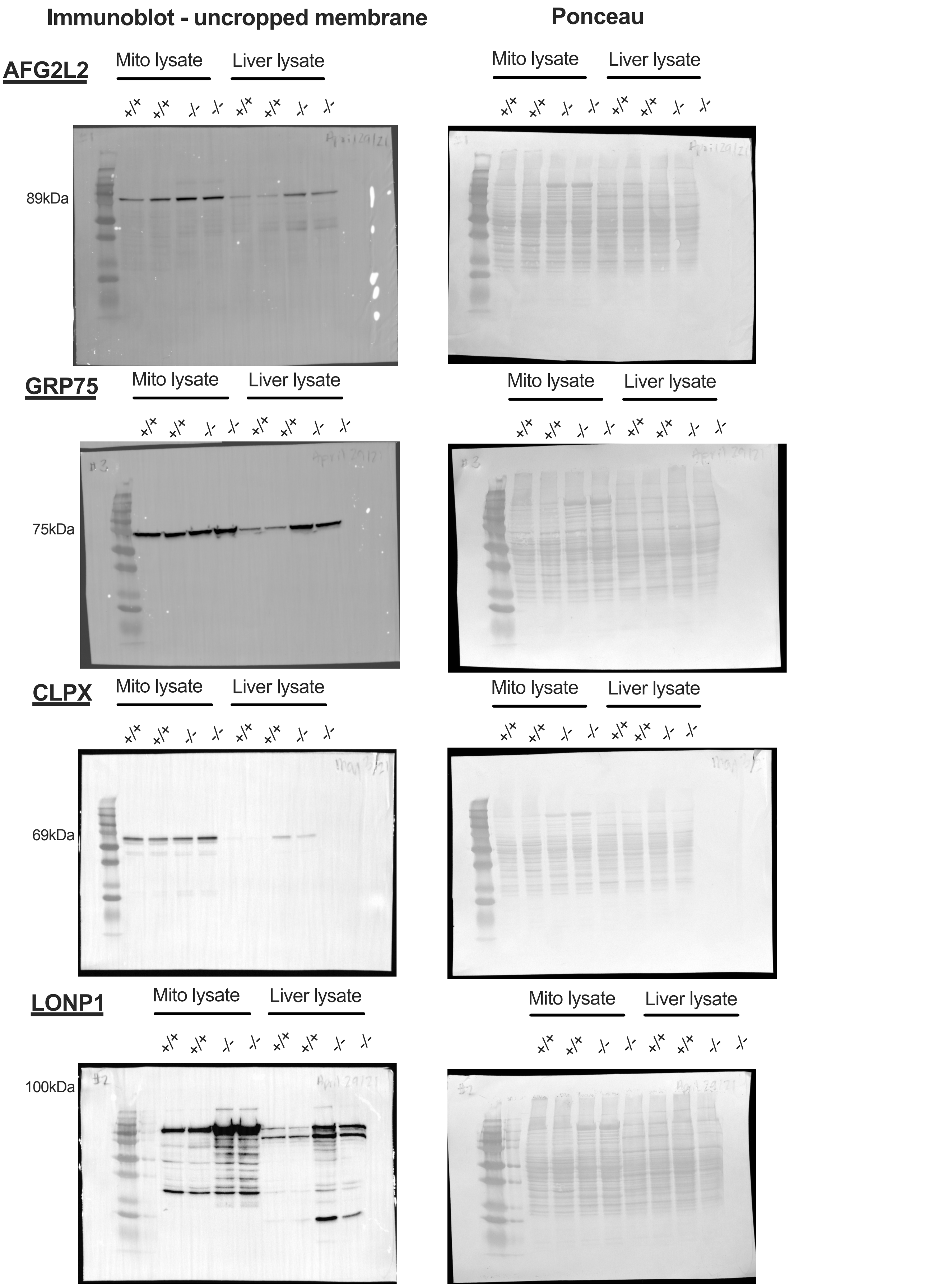

Fig S6B: Uncropped immunoblot membrane and Ponceau staining comparing mitochondrial proteases/ chaperones content in whole liver vs whole mitochondrial lysates.

Immunoblot - uncropped membrane

PGC1-α

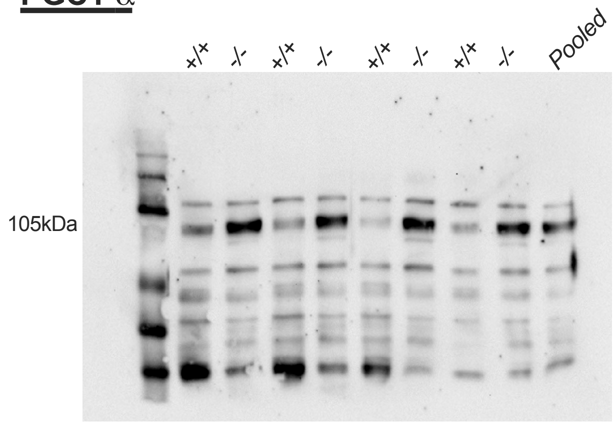

PGC1-α

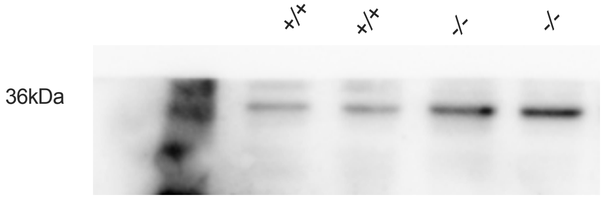

COX7A2L

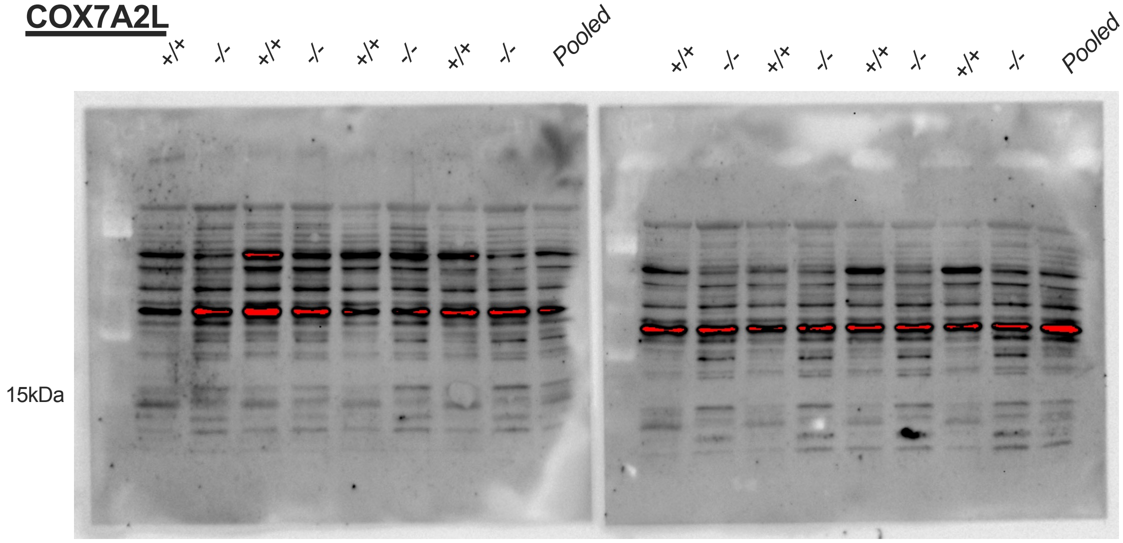

LONP

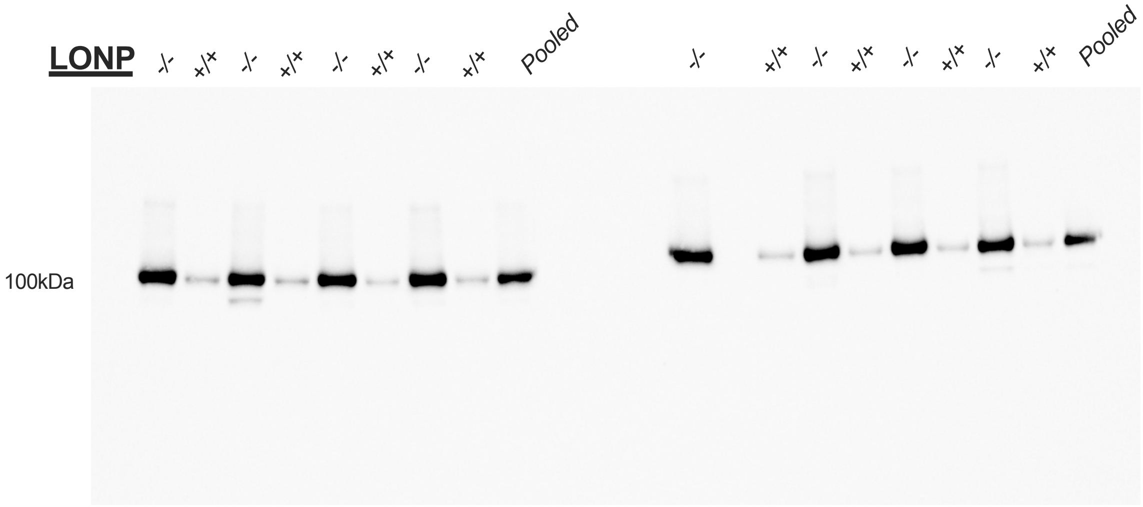

Fig S6C: Uncropped immunoblot membranes used for immunoblot experiments reported in the indicated figures.

Immunoblot - uncropped membrane

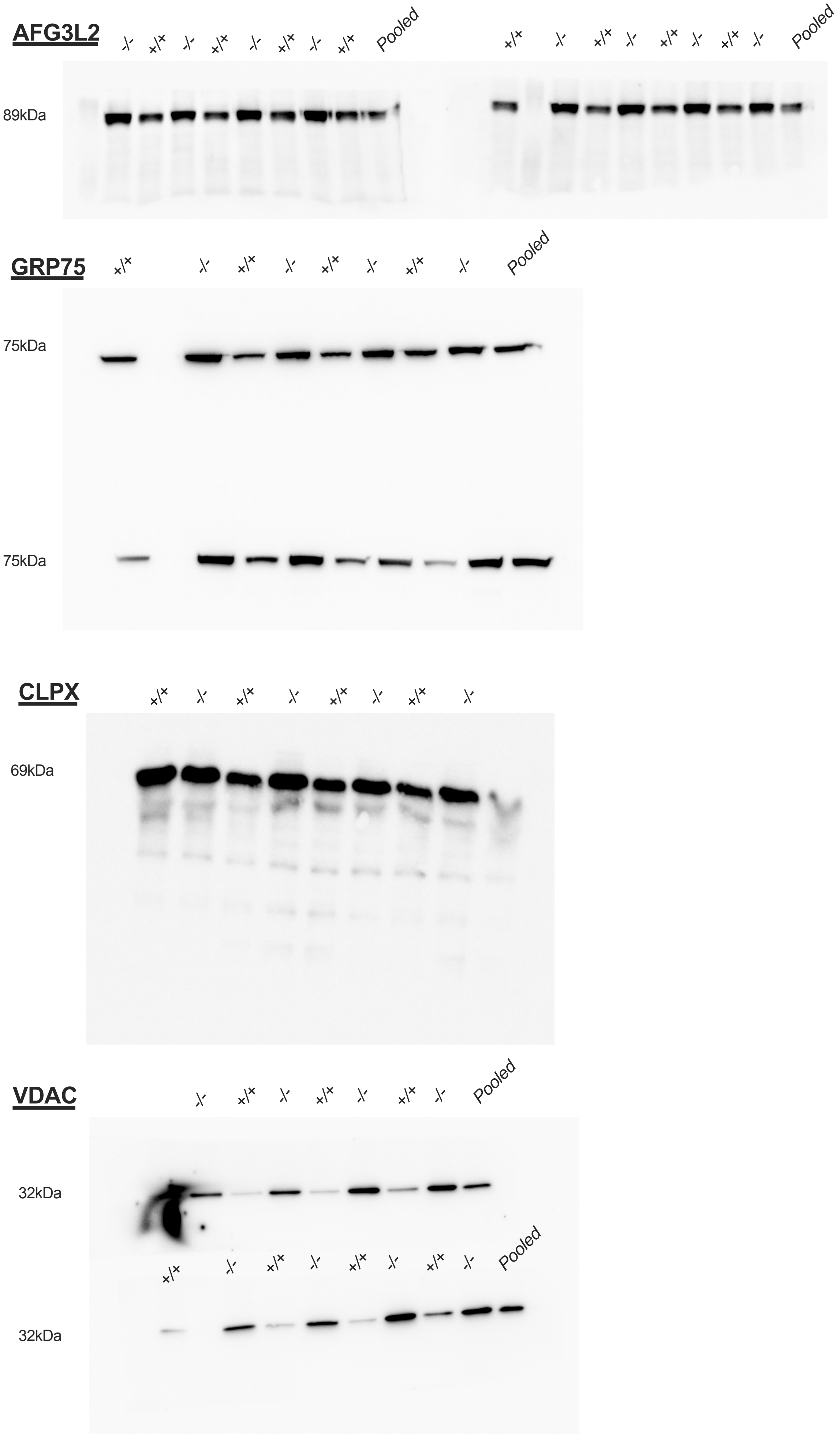

Fig S6C: Uncropped immunoblot membranes used for immunoblot experiments reported in the indicated figures.
