# Supplementary material for: Adaptive optimization of the OXPHOS assembly line partially compensates lrpprc-dependent mitochondrial translation defects in mice
Source: Commun Biol. 2021 Aug 19;4:989. doi: 10.1038/s42003-021-02492-5 (PMC8376967; doi:10.1038/s42003-021-02492-5)
Supplement: Supplementary file 5 — Reporting Summary [file 42003_2021_2492_MOESM5_ESM.pdf]

nature research

Corresponding author(s): Yan Burelle
Last updated by author(s): 2021--3-31

# Reporting Summary

Nature Research wishes to improve the reproducibility of the work that we publish. This form provides structure for consistency and transparency in reporting. For further information on Nature Research policies, see our Editorial Policies and the Editorial Policy Checklist.

## Statistics

For all statistical analyses, confirm that the following items are present in the figure legend, table legend, main text, or Methods section.

| n/a | Confirmed | |
|---|---|---|
| ☐ | ☒ | The exact sample size ($n$) for each experimental group/condition, given as a discrete number and unit of measurement |
| ☐ | ☒ | A statement on whether measurements were taken from distinct samples or whether the same sample was measured repeatedly |
| ☐ | ☒ | The statistical test(s) used AND whether they are one- or two-sided<br>*Only common tests should be described solely by name; describe more complex techniques in the Methods section.* |
| ☒ | ☐ | A description of all covariates tested |
| ☐ | ☒ | A description of any assumptions or corrections, such as tests of normality and adjustment for multiple comparisons |
| ☐ | ☒ | A full description of the statistical parameters including central tendency (e.g. means) or other basic estimates (e.g. regression coefficient) AND variation (e.g. standard deviation) or associated estimates of uncertainty (e.g. confidence intervals) |
| ☐ | ☒ | For null hypothesis testing, the test statistic (e.g. $F$, $t$, $r$) with confidence intervals, effect sizes, degrees of freedom and $P$ value noted<br>*Give P values as exact values whenever suitable.* |
| ☒ | ☐ | For Bayesian analysis, information on the choice of priors and Markov chain Monte Carlo settings |
| ☒ | ☐ | For hierarchical and complex designs, identification of the appropriate level for tests and full reporting of outcomes |
| ☒ | ☐ | Estimates of effect sizes (e.g. Cohen's $d$, Pearson's $r$), indicating how they were calculated |

*Our web collection on statistics for biologists contains articles on many of the points above.*

## Software and code

Policy information about availability of computer code

| | |
|---|---|
| Data collection | NA |
| Data analysis | NA |

For manuscripts utilizing custom algorithms or software that are central to the research but not yet described in published literature, software must be made available to editors and reviewers. We strongly encourage code deposition in a community repository (e.g. GitHub). See the Nature Research guidelines for submitting code & software for further information.

## Data

Policy information about availability of data

All manuscripts must include a data availability statement. This statement should provide the following information, where applicable:

- Accession codes, unique identifiers, or web links for publicly available datasets
- A list of figures that have associated raw data
- A description of any restrictions on data availability

The mass spectrometry proteomics data have been deposited to the ProteomeXchange Consortium via the PRIDE 79 partner repository with the dataset identifier PXD021867. All other data are available from the corresponding authors upon reasonable request.

# Field-specific reporting

Please select the one below that is the best fit for your research. If you are not sure, read the appropriate sections before making your selection.

☒ Life sciences ☐ Behavioural & social sciences ☐ Ecological, evolutionary & environmental sciences

For a reference copy of the document with all sections, see nature.com/documents/nr-reporting-summary-flat.pdf

# Life sciences study design

All studies must disclose on these points even when the disclosure is negative.

| | |
|---|---|
| Sample size | All experiments used a minimum of 3 separate experimental replicate (mice) per group. |
| Data exclusions | Results from all successful experiments were included in the study. By successful we mean that there were no obvious technical problems associated with the experiment. |
| Replication | All experiments involving proteomics and lipidomics were performed in a minimum of 3-4 distinct mice per experimental group. For functional, immunoblt and QPCR studies, 6-8 mice per group were used. |
| Randomization | Allocation was based on genotype of experimental mice (WT or Lrpprc-KO). there is no randomization |
| Blinding | Blinding to group was not possible for tissue collection and functional analyses as KO mice are discernable by eye.<br>Lipidomics and proteomics samples were coded and sent to the core facilities. there analysis was therefore blinded. |

# Reporting for specific materials, systems and methods

We require information from authors about some types of materials, experimental systems and methods used in many studies. Here, indicate whether each material, system or method listed is relevant to your study. If you are not sure if a list item applies to your research, read the appropriate section before selecting a response.

## Materials & experimental systems

| n/a | Involved in the study | |
|---|---|---|
| ☐ | ☒ | Antibodies |
| ☐ | ☒ | Eukaryotic cell lines |
| ☐ | ☐ | Palaeontology and archaeology |
| ☐ | ☒ | Animals and other organisms |
| ☐ | ☐ | Human research participants |
| ☐ | ☐ | Clinical data |
| ☐ | ☐ | Dual use research of concern |

## Methods

| n/a | Involved in the study | |
|---|---|---|
| ☐ | ☐ | ChIP-seq |
| ☐ | ☐ | Flow cytometry |
| ☐ | ☐ | MRI-based neuroimaging |

## Antibodies

| | |
|---|---|
| Antibodies used | NDUFA9 (CI, Invitrogen, #459100); SDHA (CII, Abcam, #ab14715); UQCRC2 (CIII, Abcam, #ab14745); COX4 (CIV, Invitrogen, #459600), ATPB (CV, Abcam, #ab14730), and COX7A2L (Abcam #ab66107). |
| Validation | NA |

## Eukaryotic cell lines

Policy information about cell lines

| | |
|---|---|
| Cell line source(s) | LSFC consortium Biobank Chicoutimi Quebec |
| Authentication | These are primary immortalized fibroblasts samples from LSFC patients and healthy donors. |
| Mycoplasma contamination | confirmed negative based on a monthly spot check of the cell culture facility |
| Commonly misidentified lines (See ICLAC register) | NA |

## Palaeontology and Archaeology

| | |
|---|---|
| Specimen provenance | NA |
| Specimen deposition | NA |
| Dating methods | NA |

☐ Tick this box to confirm that the raw and calibrated dates are available in the paper or in Supplementary Information.

| | |
|---|---|
| Ethics oversight | NA |

Note that full information on the approval of the study protocol must also be provided in the manuscript.

## Animals and other organisms

Policy information about studies involving animals; ARRIVE guidelines recommended for reporting animal research

| | |
|---|---|
| Laboratory animals | Hep-Lrpprc loxP/loxPxAlb-cre/cre mice, males and females, 5 weeks old |
| Wild animals | NA |
| Field-collected samples | NA |
| Ethics oversight | All experiments on animals were approved by the University of Ottawa Institutional Animal Care Committee and conducted according to the directives of the Canadian Council on Animal Care. Protocol numbers were ME-2733 and 2734. |

Note that full information on the approval of the study protocol must also be provided in the manuscript.

## Human research participants

Policy information about studies involving human research participants

| | |
|---|---|
| Population characteristics | NA |
| Recruitment | NA |
| Ethics oversight | NA |

Note that full information on the approval of the study protocol must also be provided in the manuscript.

## Clinical data

Policy information about clinical studies

All manuscripts should comply with the ICMJE guidelines for publication of clinical research and a completed CONSORT checklist must be included with all submissions.

| | |
|---|---|
| Clinical trial registration | NA |
| Study protocol | NA |
| Data collection | NA |
| Outcomes | NA |

## Dual use research of concern

Policy information about dual use research of concern

### Hazards

Could the accidental, deliberate or reckless misuse of agents or technologies generated in the work, or the application of information presented in the manuscript, pose a threat to:

| No | Yes | |
|---|---|---|
| ☒ | ☐ | Public health |
| ☒ | ☐ | National security |
| ☒ | ☐ | Crops and/or livestock |
| ☒ | ☐ | Ecosystems |
| ☒ | ☐ | Any other significant area |

### Experiments of concern

Does the work involve any of these experiments of concern:

| No | Yes | |
|---|---|---|
| ☒ | ☐ | Demonstrate how to render a vaccine ineffective |
| ☒ | ☐ | Confer resistance to therapeutically useful antibiotics or antiviral agents |
| ☒ | ☐ | Enhance the virulence of a pathogen or render a nonpathogen virulent |
| ☒ | ☐ | Increase transmissibility of a pathogen |
| ☒ | ☐ | Alter the host range of a pathogen |
| ☒ | ☐ | Enable evasion of diagnostic/detection modalities |
| ☒ | ☐ | Enable the weaponization of a biological agent or toxin |
| ☒ | ☐ | Any other potentially harmful combination of experiments and agents |

## ChIP-seq

### Data deposition

☐ Confirm that both raw and final processed data have been deposited in a public database such as GEO.

☐ Confirm that you have deposited or provided access to graph files (e.g. BED files) for the called peaks.

| | |
|---|---|
| Data access links<br>*May remain private before publication.* | NA |
| Files in database submission | NA |
| Genome browser session<br>(e.g. UCSC) | NA |

### Methodology

| | |
|---|---|
| Replicates | NA |
| Sequencing depth | NA |
| Antibodies | NA |
| Peak calling parameters | NA |
| Data quality | NA |
| Software | NA |

## Flow Cytometry

### Plots

Confirm that:

☐ The axis labels state the marker and fluorochrome used (e.g. CD4-FITC).

☐ The axis scales are clearly visible. Include numbers along axes only for bottom left plot of group (a 'group' is an analysis of identical markers).

☐ All plots are contour plots with outliers or pseudocolor plots.

☐ A numerical value for number of cells or percentage (with statistics) is provided.

### Methodology

| | |
|---|---|
| Sample preparation | NA |
| Instrument | NA |
| Software | NA |
| Cell population abundance | NA |
| Gating strategy | NA |

☐ Tick this box to confirm that a figure exemplifying the gating strategy is provided in the Supplementary Information.

## Magnetic resonance imaging

### Experimental design

| | |
|---|---|
| Design type | NA |
| Design specifications | NA |
| Behavioral performance measures | NA |

### Acquisition

| | |
|---|---|
| Imaging type(s) | NA |
| Field strength | NA |
| Sequence & imaging parameters | NA |
| Area of acquisition | NA |

Diffusion MRI ☐ Used ☐ Not used

### Preprocessing

| | |
|---|---|
| Preprocessing software | NA |
| Normalization | NA |
| Normalization template | NA |
| Noise and artifact removal | NA |
| Volume censoring | NA |

### Statistical modeling & inference

| | |
|---|---|
| Model type and settings | NA |
| Effect(s) tested | NA |

Specify type of analysis: ☐ Whole brain ☐ ROI-based ☐ Both

| | |
|---|---|
| Statistic type for inference (See Eklund et al. 2016) | NA |
| Correction | NA |

### Models & analysis

| n/a | Involved in the study |
|---|---|
| ☐ | ☐ Functional and/or effective connectivity |
| ☐ | ☐ Graph analysis |
| ☐ | ☐ Multivariate modeling or predictive analysis |

| | |
|---|---|
| Functional and/or effective connectivity | NA |
| Graph analysis | NA |
| Multivariate modeling and predictive analysis | NA |
